# Immunogenicity and safety of three consecutive production lots of the non replicating smallpox vaccine MVA: A randomised, double blind, placebo controlled phase III trial

**DOI:** 10.1371/journal.pone.0195897

**Published:** 2018-04-13

**Authors:** Edgar Turner Overton, Steven J. Lawrence, Eva Wagner, Katrin Nopora, Siegfried Rösch, Philip Young, Darja Schmidt, Christian Kreusel, Sonja De Carli, Thomas P. Meyer, Heinz Weidenthaler, Nathaly Samy, Paul Chaplin

**Affiliations:** 1 Division of Infectious Diseases, University of Alabama at Birmingham School of Medicine, Birmingham, Alabama, United States of America; 2 Division of Infectious Diseases, Washington University School of Medicine, St. Louis, Missouri, United States of America; 3 Bavarian Nordic GmbH, Martinsried, Germany; Beth Israel Deaconess Medical Center, UNITED STATES

## Abstract

**Background:**

Modified Vaccinia Ankara (MVA) is a live, viral vaccine under advanced development as a non-replicating smallpox vaccine. A randomised, double-blind, placebo-controlled phase III clinical trial was conducted to demonstrate the humoral immunogenic equivalence of three consecutively manufactured MVA production lots, and to confirm the safety and tolerability of MVA focusing on cardiac readouts.

**Methods:**

The trial was conducted at 34 sites in the US. Vaccinia-naïve adults aged 18-40 years were randomly allocated to one of four groups using a 1:1:1:1 randomization scheme. Subjects received either two MVA injections from three consecutive lots (Groups 1-3), or two placebo injections (Group 4), four weeks apart. Everyone except personnel involved in vaccine handling and administration was blinded to treatment. Safety assessment focused on cardiac monitoring throughout the trial. Vaccinia-specific antibody titers were measured using a Plaque Reduction Neutralization Test (PRNT) and an Enzyme-Linked Immunosorbent Assay (ELISA). The primary immunogenicity endpoint was Geometric Mean Titers (GMTs) after two MVA vaccinations measured by PRNT at trial visit 4. This trial is registered with ClinicalTrials.gov, number NCT01144637.

**Results:**

Between March 2013 and May 2014, 4005 subjects were enrolled and received at least one injection of MVA (n = 3003) or placebo (n = 1002). The three MVA lots induced equivalent antibody titers two weeks after the second vaccination, with seroconversion rates of 99·8% (PRNT) and 99·7% (ELISA). Overall, 180 (6·0%) subjects receiving MVA and 29 (2·9%) subjects in the placebo group reported at least one unsolicited Adverse Event (AE) that was considered trial-related. Vaccination was well tolerated without significant safety concerns, particularly regarding cardiac assessment.

**Conclusions:**

The neutralizing and total antibody titers induced by each of the three lots were equivalent. No significant safety concerns emerged in this healthy trial population, especially regarding cardiac safety, thus confirming the excellent safety and tolerability profile of MVA.

**Trial registration:**

ClinicalTrials.gov NCT01144637

## Introduction

Despite the eradication of smallpox in 1980 [1], the risk of reoccurrence still remains due to its potential use in biological warfare [2, 3]. The limited immunity in the general population due to the discontinuation of routine vaccination programs raises the need for contingency plans involving vaccine stockpiles. However, current stockpiles comprise traditional replicating vaccines that are contraindicated for up to 25% of the population (e.g. individuals with impaired immune systems or eczematous skin disease, as well as their contacts) [4]. Furthermore, a series of cardiac adverse events, including myo-/pericarditis, have been observed in close temporal relationship to the administration of replicating smallpox vaccines such as ACAM2000 [5–9]. Their use in a pre-emergency situation is thus restricted to high-risk populations (e.g. military personnel), disregarding other relevant groups such as first-line responders. Safer vaccines are thus needed to encompass a wider population. MVA is a live, highly attenuated Modified Vaccinia Ankara virus currently in advanced clinical development as a non-replicating smallpox vaccine and is considered to be a safer alternative over replicating smallpox vaccines [10].

MVA has demonstrated protective efficacy in animal models, including non-human primates [11], as well as robust immunogenicity and a good safety profile in healthy individuals, including elderly and at-risk populations usually contraindicated to receive replicating smallpox vaccines [people infected with human immunodeficiency virus (HIV), diagnosed with atopic dermatitis (AD), or under immune suppressive treatment] [12–17]. Moreover, MVA induces peak antibody responses comparable to the ones elicited by the replicating smallpox vaccine Dryvax^®^ in healthy subjects. Prior MVA vaccination inhibits the replication of vaccinia virus (Dryvax^®^) in the skin [18]. In order to further address the potential efficacy of MVA as a reliable inoculation strategy against smallpox, clinical trials performed so far have included vaccinia-naïve as well as vaccinia-experienced subjects [12,13,15,19]. In 2010, MVA was granted a pre-emergency use authorization status in the U.S. for the vaccination of vulnerable populations contraindicated to receive replicating smallpox vaccines, such as HIV-infected individuals and subjects with atopic dermatitis (AD). MVA has been licensed in the European Union (EU) and Canada since 2013 (trade name outside EU IMVAMUNE^®^; invented name in the EU IMVANEX^®^) [17,18,20,21].

The objectives of the phase III trial reported here were to demonstrate the equivalence of three consecutive MVA production lots in terms of induced antibody titers, and to confirm the favorable cardiac safety and tolerability profile of MVA.

## Methods

### Trial design and participants

This randomised, double-blind, placebo-controlled Phase III trial was conducted at 34 sites in the US between March 18, 2013 and May 23, 2014. The study was approved by the following Institutional Review Boards: Quorum Review IRB, 1601 5^th^ Avenue, Seattle, WA; Washington University in St. Louis Human Research Protective Office, 600 South Euclid Avenue, St. Louis, MO; Marshall University IRB #1 / Office of Research Integrity, 401 11^th^ Street, Huntington, WV; The University of Alabama at Birmingham IRB for Human Use, 701 20^th^ Street South, Birmingham, AL.

Healthy, vaccinia-naïve, women and men aged 18-40 years were randomised into four groups: Groups 1 to 3 received two subcutaneous (s.c.) MVA injections from three different consecutively manufactured vaccine lots at weeks 0 and 4. Group 4 received two s.c. placebo injections in the same time interval. The active trial period (8-10 weeks) comprised five visits. A phone follow-up (FU) visit was performed 26 weeks after the second injection. The trial was conducted according to ICH-GCP standards and the principles of the Declaration of Helsinki. The protocol was approved by independent review committees for each site and all subjects provided written informed consent prior to participation.

### Randomisation and blinding

Treatment was assigned at trial visit 1 after re-confirmation of the subject’s eligibility. The randomisation scheme was 1:1:1:1 (3 MVA groups and 1 placebo group). Randomisation was stratified by clinical trial site. The unblinded trial statistician generated randomisation sequences (block randomisation with block size of eight) and the unblinded clinical trial staff assigned each participant to a treatment group using a secure interactive web response system.

Everyone except personnel performing randomization, vaccine handling and administration, remained blinded to treatment throughout the study.

### Procedures

The MVA bulk drug substance was produced at Bavarian Nordic A/S (Kvistgard, Denmark) according to cGMP standard and the final drug product was filled, formulated and labelled at IDT Biologika GmbH (Dessau-Roßlau, Germany). One dose (0·5 mL) of the liquid frozen formulation had a nominal virus titre of 1 x 10^8^ TCID_50_. Placebo consisted of the vaccine formulation buffer, Tris-buffered saline (TBS), provided in liquid aliquots. One placebo dose (0·5 mL) contained 0·605 mg tris (hydroxymethyl)-amino methane and 4·09 mg sodium chloride.

To evaluate the safety of MVA, solicited and unsolicited AEs were recorded at all visits. Serious Adverse Events (SAEs) were monitored up to the six months phone FU visit. Solicited AEs constituted a set of pre-defined, expected local reactions (erythema, swelling, pain, itching, and induration) and systemic symptoms (pyrexia, headache, myalgia, nausea, fatigue and chills). All solicited AEs were recorded on a memory aid during an 8-day period following each injection. Treatment-emergent unsolicited AEs were events not included in the memory aid and reported by the subject within a 29-day period after each injection. Safety laboratory tests were performed at screening and two weeks after each injection. Troponin I testing and electrocardiograms (ECG) were performed at screening and two weeks after the first injection. If clinically indicated, additional safety measures could be taken at any other scheduled or unscheduled visit. Any cardiac symptoms developed since the first injection, clinically significant ECG changes and Troponin I elevations ≥2 x upper limit of normal (ULN) were defined as AEs of special interest (AESIs). Any subject developing an AESI was requested to return to the clinical trial site in order to perform a thorough work-up, physical and cardiac examination comprising ECG, cardiac enzymes and/or echocardiogram. If necessary, further diagnostic tests and regular follow-ups could be performed until complete resolution or stabilization.

Blood for immunogenicity serum collection was drawn at baseline prior to the first injection (week 0) and two weeks after the second injection (week 6). These time points are based on results obtained during the phase I and II development program, in which peak antibody titers were consistently observed two weeks after the second vaccination in vaccinia-naïve subjects [15,16,19–22]. PRNT and ELISA were performed as previously described with the following modifications: For the ELISA, a cut off value of 0·35 was used. For the PRNT, the neutralization was performed in Dulbecco’s modified Eagle’s medium/0·1% human serum albumin and the detection limit was a titer of 2 [15]. Seroconversion was defined as the appearance of titers equal to or higher than the detection limit for subjects seronegative at baseline, or as an at least 2-fold increase over pre-existing titers for subjects tested positive at baseline.

## Objectives

The primary objective was to assess the equivalence of three consecutively produced MVA lots by PRNT. The secondary objectives were to assess uncommon adverse reactions, with a particular focus on cardiac signs and symptoms indicating myo- /pericarditis, and to compare their frequency to those observed after placebo. Additionally, vaccinia-specific humoral immune evaluations utilizing an ELISA and a correlation analysis between total and neutralizing antibody titers were performed.

### Criteria for evaluation

**Primary immunogenicity endpoint**:

GMTs after two MVA vaccinations measured by PRNT at trial visit 4.

**Secondary immunogenicity endpoints**:

1. GMTs after two MVA vaccinations measured by ELISA at trial visit 4.
2. Vaccinia-specific PRNT and ELISA seroconversion rates at trial visit 4
3. Pearson Correlation Coefficient between the log_10_ transformed PRNT titers and the log_10_ transformed ELISA titers at trial visit 4.

**Secondary safety and reactogenicity endpoints**:

1. Occurrence, relationship and intensity of any Serious Adverse Event (SAE) at any time during the trial.
2. Occurrence, relationship and intensity of any cardiac sign or symptom indicating a case of myo-/pericarditis
3. Occurrence of any Grade 3 or 4 Adverse Events (AEs) probably, possibly or definitely related to the trial vaccine within 28 days after vaccination.
4. Occurrence, relationship and intensity of unsolicited non-serious AEs within 28 days after each vaccination.
5. Occurrence, relationship and intensity of solicited local AEs (erythema, swelling, pain, itching, and induration) during the 8-day period (day of vaccination and the following seven days) after each vaccination.
6. Occurrence, relationship, intensity and duration of solicited general AEs (pyrexia, headache, myalgia, nausea, fatigue and chills) during the 8-day period (day of vaccination and the following seven days) after each vaccination.

#### Datasets analysed

The Full Analysis Set (FAS) consisted of all randomized subjects who received at least one vaccination and for whom any data were available. For all analyses using the FAS, subjects were summarized according to the treatment that they received.

The Immunogenicity Analysis Set (IAS) was a subset of the FAS comprised of the first 700 randomized subjects from each group.

The Per Protocol Set (PPS) consisted of all subjects present in the IAS who received both vaccinations, completed Visits 1, 3, and 4, and adhered to all protocol conditions. Subjects with only minor protocol deviations were included in this dataset.

### Statistical analysis

Statistical analyses were performed using SAS version 9.1 (SAS Institute, Cary, NC).

Assuming a significance level of 5%, an analyzable sample size of 600 per group for the PPS, and an expected log_10_ titer SD of 0.85 in all groups receiving MVA for the PRNT, the primary hypothesis of the PRNT GMT at Visit 4 (i.e., after two MVA vaccinations) has a power of > 80% to show equivalence for all three MVA groups, using an equivalence margin of Δ = 0.301 on the log_10_ scale. In order to account for a dropout rate of about 15%, which has been observed in previous MVA trials, serum collection was scheduled for a total of 700 subjects in each group.

To allow a proper safety analysis, 300 additional subjects were enrolled in each group to ensure that a total of ~ 1,000 subjects per group were available, giving a combined MVA safety population of 3,000 subjects. With this sample size the trial had a 95% chance of detecting any uncommon AEs, i.e., AEs with an incidence of at least 1/1,000.

Immunogenicity analyses were primarily ran on the PPS and the IAS.

The PRNT and ELISA titers were transformed into log_10_ titers for the calculation of geometric means and 95% confidence intervals (CIs). Titers below the detection limit were assigned a value of 1 for GMT calculation.

For lot equivalence, a two-sided 95% CI was calculated using the differences of the means of the log_10_-transformed post baseline titers between pairs of lots. Equivalence among the three lots was demonstrated if for each pair of lots, the two-sided 95% CI for the log_10_ PRNT mean was between -0·301 and 0·301, i.e., if the PRNT GMT ratio of each pair of lots was between 0·5 and 2·0. For the ELISA, the two-sided 95% CI was -0·176 and 0·176, i.e. the ELISA GMT ratio was between 0·66 and 1·5.

For the PRNT and ELISA correlation, Pearson’s correlation coefficient (with associated CI) between log_10_ titers measured by both assays was calculated per group and for Groups 1–3 combined along with the associated p-values and 95% CIs.

The trial was overseen by an independent data safety monitoring board (DSMB; 4 meetings) and is registered with ClinicalTrials.gov, number NCT01144637.

## Results

**Fig 1:**
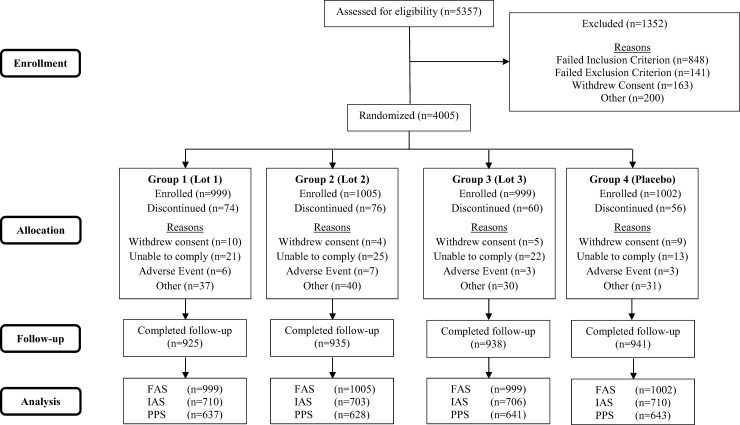
Subject disposition. FAS = full analysis set; IAS = Immunogenicity Analysis Set, subset used for immunogenicity analysis (first ~ 700 subjects enrolled per group); PPS = per protocol set; n = number of subjects in the specified category.

A total of 5357 volunteers aged 18-40 years were screened, of whom 4005 were eligible for enrolment. All eligible subjects received at least one injection, had data available for analysis and were included in the FAS. Of these 4005 subjects, 2829 were included in the IAS. Of the 2829 subjects in the IAS, 2549 were eligible for the PPS (Fig 1).

There were no statistically significant differences for any demographic characteristics among treatment groups (Table 1).

**Table 1:** Demographic data (FAS, n = 4005).

Group		Group 1 (Lot 1) (n = 999)	Group 2 (Lot 2) (n = 1005)	Group 3 (Lot 3) (n = 999)	Groups 1–3 (n = 3003)	Group 4 (Placebo) (n = 1002)
Age [years]	Mean	28	27	28	28	28
	SD	6·3	6·2	6·3	6·3	6·4
Height [cm]	Mean	170	171	171	171	171
	SD	9·6	10·1	9·6	9·7	10·1
Weight [kg]	Mean	76	77	77	77	76
	SD	14·8	15·5	15·4	15·2	15·8
Gender	Male	473 (47%)	478 (48%)	505 (51%)	1456 (48%)	463 (46%)
	Female	526 (53%)	527 (52%)	494 (49%)	1547 (52%)	539 (54%)
Ethnicity	Hispanic/Latino	119 (12%)	119 (12%)	108 (11%)	346 (12%)	109 (11%)
	Non-Hispanic/Latino	880 (88%)	886 (88%)	890 (89%)	2656 (88%)	893 (89%)
Race	White/Caucasian	773 (78%)	790 (79%)	765 (77%)	2328 (77%)	773 (77%)
	Black/African American	172 (17%)	165 (16%)	191 (19%)	528 (18%)	184 (18%)
	Oriental/Asian	24 (2%)	17 (2%)	18 (2%)	59 (2%)	19 (2%)
	Other	30 (3%)	33 (3%)	25 (2%)	88 (3%)	26 (3%)

Data for gender, ethnicity and race are n (%), SD = standard deviation.

Vaccinations were generally well tolerated.

Higher proportions of subjects vaccinated with MVA experienced solicited local AEs compared to Placebo (Group 1: 892/985 [90·6%]; Group 2: 865/976 [88·6%]; Group 3: 879/982 [89·5%] and Placebo: 344/980 [35·1%] as well as solicited general AEs compared to Placebo (Group 1: 588/985 [59·7%]; Group 2: 577/976 [59·1%]; Group 3: 589/982 [60·0%] and Placebo: 381/980 [38·9%]).

Table 2 presents an overview of the incidence of all unsolicited AEs. A total of 180 (6·0%) subjects in the combined MVA groups (Groups 1–3) reported at least 1 unsolicited AE that was considered by the investigator to be (at least possibly) related to the trial vaccine. A smaller proportion of subjects in the placebo group (2·9%) experienced related unsolicited AEs (p = 0·0001).

**Table 2:** Overview of unsolicited adverse events per subject (FAS, n = 4005).

	Group 1 (Lot 1) (n = 999)	Group 2 (Lot 2) (n = 1005)	Group 3 (Lot 3) (n = 999)	Group 1–3 (n = 3003) p-value^#^	Group 4 (Placebo) (n = 1002)
SAE	11 (1·1)*	7 (0·7)	7 (0·7)	25 (0·8) p = 1·0000	8 (0·8)
At least possibly related SAE	0 (0·0)	0 (0·0)	0 (0·0)	0 (0·0) p = 0·2502	1 (0·1)
AESI	2 (0·2)	5 (0·5)	1 (0·1)	8 (0·3) p = 0·4654	1 (0·1)
At least possibly related AESI	0 (0·0)	1 (0·1)	1 (0·1)	2 (0·1) p = 1·0000	0 (0·0)
Unsolicited AE	199 (19·9)	238 (23·7)	223 (22·3)	660 (22·0) p = 0·0400	189 (18·9)
At least possibly related unsolicited AE	54 (5·4)	59 (5·9)	67 (6·7)	180 (6·0) p<0·0001	29 (2·9)
At least possibly related unsolicited AE Grade0020030≥ 3	2 (0·2)	2 (0·2)	2 (0·2)	6 (0·2) p = 0·6882	1 (0·1)
AE leading to withdrawal from trial	5 (0·6)	9 (0·8)	4 (0·5)	18 (0·6) p = 0·3202	3 (0·3)

Data are n (%)

* One subject in Group 1 committed suicide 19 days after receiving the first injection. The investigator assessed this death as unrelated to treatment.

# p-value calculated using Fisher’s exact test comparing the incidences in the combined Groups 1–3 versus Group 4

Injection site induration and injection site hematoma were the only unsolicited AEs experienced by more than 1% of the subjects and mostly occurred after the first vaccine administration (data not shown).

A total of 18/3003 MVA-vaccinated subjects (0·6%) and 3/1002 placebo-vaccinated subjects (0·3%) were withdrawn from the trial due to an AE, most of which were assessed as unrelated or unlikely related to the trial vaccine, and had a mild to moderate intensity. Four subjects receiving MVA were withdrawn due to AEs corresponding to arthralgia, bundle branch block, pericarditis or pruritus, assessed as possibly related to the trial vaccine. The occurrence of two life-threatening events (a suicide and a suicide attempt) leading to withdrawal of subjects receiving MVA was assessed as unrelated to the vaccine. One subject in the placebo group experienced a seizure in temporal relation to the trial vaccination. This possibly related SAE of moderate intensity was the only Suspected Unexpected Serious Adverse Reaction (SUSAR) occurring during the trial. Injection site reactions and an AE of dizziness leading to withdrawal were assessed as definitely related to the vaccine. Overall, the AE safety profile across the three MVA groups was similar (Table 2).

A small proportion of subjects (< 1%) in the placebo group and the combined MVA groups experienced SAEs (p > 0·9999).

A total of 8/3003 subjects (0·3%) in the combined MVA groups experienced AESI compared to one subject in the placebo group (Table 2). Two subjects with elevated Troponin I values (0·13 ng/mL and 0·14 ng/mL) and no abnormal ECG findings returned to normal levels upon retest. Neither was deemed as related to vaccine. Furthermore, five subjects (four in the MVA groups and one in the placebo group) were referred to a cardiologist for evaluation without apparent indication of cardiac toxicity.

Two out of the 3003 MVA-vaccinated subjects (0·1%) experienced AESI related to MVA (Table 2): one in Group 2 exhibited a right bundle branch block in the ECG and another in Group 3 experienced symptoms indicating possible acute pericarditis according to protocol criteria (chest pain worsening when lying down) (Table 3). A thorough cardiac examination, including auscultation, ECG, Troponin I testing and echocardiography did not confirm the diagnosis. The echocardiography did not reveal any signs of pericardial effusion, pericardial rub, ECG changes suggestive of pericarditis, Troponin I increase or decreased exercise capacity. A detailed laboratory examination revealed a positive serology for Coxsackie B virus in temporal relation to the reported chest pain, suggesting a possible acute viral infection as the potential cause of the symptoms.

**Table 3:** Adverse events of special interest (FAS, n = 4005).

	Preferred term	Relationship to trial vaccine determined by investigator	Outcome
**Group 1 (Lot 1)**			
	Troponin I increase ≥ 2 x ULN	Unlikely	Resolved
	Wolff-Parkinson-White Syndrome	Unrelated	Resolved
**Group 2 (Lot 2)**			
	Troponin I increase ≥ 2 x ULN	Unlikely	Resolved
	Supraventricular extrasystoles	Unrelated	Resolved
	Bundle branch block right	Possible	Not Resolved
	Tachycardia	Unrelated	Resolved
	ECG ST Segment abnormal	Unrelated	Unknown
**Group 3 (Lot 3)**			
	Pericarditis	Possible	Resolved
**Group 4 (Placebo)**			
	Bundle branch block right	Unrelated	Resolved

No subjects in the placebo group experienced AESI considered related to trial vaccine. All AESIs, except a tachycardia experienced by a subject in Group 2, occurred after the first vaccination.

Immunogenicity data for the PPS are shown in Table 4. Similar results were obtained for the IAS. Comparable baseline titers were observed amongst the groups (PRNT: GMT of 1·0 for all groups; ELISA: GMT ranging from 1·1 to 1·3; data not shown) indicating no baseline immunity to smallpox. Two weeks after the second vaccination, vaccinia-specific neutralizing (PRNT) titers had risen to 110·7, 110·5 and 117·2 in Groups 1, 2 and 3 respectively, while the placebo Group had a GMT of 1·0. For total antibodies, ELISA GMT values of 901·0, 794·4 and 946·7 were observed for Groups 1, 2 and 3 respectively, with a GMT of 1·1 in the placebo Group. In both the PRNT and ELISA, ≥99·5% of subjects had seroconverted two weeks after the second vaccination across all MVA groups, with 1·4% (PRNT) and 2·0% (ELISA) of subjects meeting the criteria for seroconversion in the placebo group. Equivalence was statistically proven with a ratio of 1·10, 0·94 and 0·86 in the PRNT for Group 1 / Group 2, Group 1 / Group 3 and Group 2 / Group 3 respectively, meeting the trial primary endpoint. Similar ratios (1·13, 0·95 and 0·84) were determined through ELISA for the respective group pairs (Table 4). PRNT and ELISA titers were significantly correlated two weeks after the second vaccination in all groups (p < 0·0001, data not shown).

**Table 4:** PRNT and ELISA GMTs, seroconversion rates and GMT group ratios 2 weeks after the second vaccination (PPS, n = 2549).

**PRNT and ELISA GMT and seroconversion rates**								
**Group**		**Group 1 (Lot 1) (n = 637)**	**Group 2 (Lot 2) (n = 628)**		**Group 3 (Lot 3) (n = 641)**	**Group 1–3 (n = 1906)**		**Group 4 (Placebo) (n = 643)**
GMT [95% CI] SC	PRNT	110·7 [103·4, 118·4] 99·8%	110·5 [93·7, 107·8] 99·7%		117·2 [109·0, 126·0] 99·8%	109·3 [104·9, 113·8] 99·8%		1·0 [1·0, 1·1] 1·4%
	ELISA	901·0 [844·8, 961·1] 99·7%	794·4 [738·1, 855·0] 99·5%		946·7 [888·2, 1008·9] 100·0%	878·9 [845·3, 913·8] 99·7%		1·1 [1·1, 1·3] 2·0%
**Group Ratios of PRNT and ELISA GMT**								
**Group Ratio**		**Group 1/Group 2**		**Group 1/Group 3**			**Group 2/Group 3**	
Ratio of GMT [95% CI] Equivalence Met*	PRNT	1·1012 [0·9992, 1·2136] Yes		0·9444 [0·8554, 1·0427] Yes			0·8577 [0·7753, 0·9488] Yes	
	ELISA	1·1342 [1·0288, 1·2505] Yes		0·9518 [0·8695, 1·0419] Yes			0·8392 [0·7615, 0·9247] Yes	

GMT: Geometric Mean Titer (for purposes of calculation titers below the detection limit are given the arbitrary value 1)

95% CI: 95% confidence interval [LCL = lower confidence limit and UCL = upper confidence limit]

SC: Seroconversion is the percentage of subjects who became seropositive or had an at least two-fold rise in titer from baseline

*: Equivalence is passed if the LCL > 1 / Delta and UCL < Delta. For the PRNT Delta is 2, i.e. CI must be contained in [1/2, 2]. For the ELISA Delta is 1·5, i.e. the CI must be contained in [2/3, 1·5].

## Discussion

This phase III trial was designed according to the recommendations issued by the US FDA to commercialize a non-replicating smallpox vaccine. The results demonstrate a consistent MVA manufacturing process and confirm the favorable cardiac safety profile of MVA in a large population.

Comparable vaccinia-specific PRNT GMT values were observed two weeks following the second vaccination (week 6) in the PPS, demonstrating the equivalence of three consecutively produced MVA lots. MVA induced strong total (ELISA) and neutralizing (PRNT) antibody responses, with seroconversion rates of at least 99**·**5% after the second vaccination. Analogous findings were previously observed using the same MVA administration scheme [14,15,20–22], showing a reliable immunogenic potential.

This trial adds a large dataset of almost 2000 paired ELISA and PRNT titers to the immunogenicity database, exhibiting a significant correlation between total and neutralizing antibody titers that confirms the results of previous clinical trials [17]. Similarly, these results significantly enlarged the overall existing safety database of MVA. The addition of 3003 MVA vaccinated subjects, together with the 651 subjects from a recently completed phase II trial (publication in preparation), has doubled the size of the MVA safety database, i.e. cumulative safety data are now available from almost 8000 vaccinees.

Most of the AEs reported after MVA administration in this trial comprised temporary local and systemic reactions of mild to moderate intensity that did not require additional treatment nor led to withdrawal from further vaccination. As expected, the frequency of solicited AEs observed in the three MVA groups was significantly higher compared to the placebo group. Their frequency, intensity and duration were equally distributed across the 3 MVA groups, showing lot equivalence. This profile was similar to those established during the phase I and II clinical trial programs, further demonstrating the excellent and reliable safety of MVA across different populations [12, 13, 20–23].

Concerning unsolicited AEs, including SAEs, no trends towards any specific events or system organ classes were observed. The overall number of unsolicited AEs and SAEs was low and the distribution was comparable across all treatment groups.

Replicating smallpox vaccines are associated with a series of cardiac AEs of inflammatory nature, such as myo/pericarditis. MVA has exhibited a lack of such effects in almost 8000 vaccinees [6, 24]. A lower incidence of AESI was observed in the present study compared to previous trials, with no significant difference between the MVA and placebo groups. No signs of cardiac inflammatory disorders were detected in the 3003 subjects treated with MVA. A single case of chest pain that worsened when lying down occurred during the trial, meeting the protocol criteria for possible acute pericarditis. However, thorough cardiac examinations revealed normal ECG results without pericardial effusion and normal Troponin I levels. This event was therefore assessed as unlikely related to MVA and resolved spontaneously with no sequelae. These findings are in line with previous studies, suggesting a robust cardiac safety profile [12, 13].

Only three of the 3003 MVA-vaccinates showed an increase of Troponin I above normal limit following vaccination. One subject exhibited an elevated Troponin I value at baseline that did not classify as an AESI and therefore met the inclusion criterion for enrolment. This value later increased above baseline levels. The second subject showed increased Troponin I levels upon the second visit (14 days after the first vaccination), accompanied by normal ECG results. His Troponin I levels returned to normal 5 days later. The event was considered as resolved and assessed as unlikely related to the trial vaccine. The third subject had elevated Troponin I levels 15 days after vaccination. Further cardiac examination revealed signs suggesting athlete’s heart, which coincided with a history of marathon running. The event was later considered as resolved and assessed as unlikely related to trial vaccine. In summary, the frequency of Troponin I increase above ULN following MVA administration was below 1 in 1000.

All AESI were transient without long-term cardiac damage according to the cardiology follow-up examinations. AESI reports were based on findings obtained through the targeted examinations including Troponin I testing and ECG readings. Epidemiologic figures for background incidence of Troponin I elevations are in the range of 0·43% to 1·5%, depending on the definition of the upper limit of normal [25–27]. The background incidence of pericarditis shows a frequency of up to 27·7 cases per 100,000 patients [28]. The incidence for all AESI reported in this trial is thus in line with or below the published background incidence rates for otherwise healthy populations, suggesting that MVA administration presents no increased cardiac risk to recipients.

This phase III trial further demonstrates the favorable cardiac safety profile of the non-replicating smallpox vaccine MVA. Replication-competent smallpox vaccines such as Dryvax and ACAM2000 are associated with a significant risk of myo-/pericarditis [6–10, 29], with incidence rates ranging from 1 in 175 to 1 in 216 vaccinees following vaccination [29, 30]. A recent prospective surveillance study reported a significant increased risk to develop cardiovascular symptoms such as chest pain (approx. 1 in 12 vaccinees) and dyspnoea (approx. 1 in 19 vaccinees) after receiving ACAM2000 [30]. In the present trial, isolated cases of chest pain/discomfort and dyspnoea were equally distributed between MVA and placebo groups, making unlikely any association between these symptoms and MVA.

The overall AE profile observed in this trial coincides with reports from previous trials [12, 13, 15, 17, 19–22].

In summary, this trial has confirmed the excellent safety and cardiac profile of MVA, clearly differentiating MVA from replicating smallpox vaccines. In addition, the robust immunogenicity displayed by the different lots indicates a reliable and consistent manufacturing process.

## Supporting information

S1 File Poster. (PDF)

S2 File Patents by Paul Chaplin. (DOCX)

S3 File Clinical trial protocol. (PDF)

S4 File CONSORT 2010 checklist. (DOC)

## References

[1] Breman JG, Arita I. The confirmation and maintenance of smallpox eradication. *N Engl J Med* 1980; 303: 1263–73. doi: 10.1056/NEJM198011273032204 6252467 10.1056/NEJM198011273032204

[2] Mayr A. Smallpox vaccination and bioterrorism with pox viruses. *Comp Immunol Microbiol Infect Dis* 2003; 26: 423–30. doi: 10.1016/S0147-9571(03)00025-0 12818626 10.1016/S0147-9571(03)00025-0

[3] Artenstein AW, Grabenstein JD. Smallpox vaccines for biodefense: need and feasibility. *Expert Rev Vaccines* 2008; 7: 1225–37. doi: 10.1586/14760584.7.8.1225 18844596 10.1586/14760584.7.8.1225 PMC9709930

[4] Lane JM, Ruben FL, Neff JM, Millar JD. Complications of smallpox vaccination, 1968: results of ten statewide surveys. *J Infect Dis* 1970; 122: 303–09. 4396189 10.1093/infdis/122.4.303

[5] McNeil MM, Cano M, Miller R, Petersen BW, Engler RJ, Bryant-Genevier MG. Ischemic cardiac events and other adverse events following ACAM2000(®) smallpox vaccine in the Vaccine Adverse Event Reporting System. *Vaccine* 2014; 32: 4758–65. doi: 10.1016/j.vaccine.2014.06.034 24951868 10.1016/j.vaccine.2014.06.034 PMC5946322

[6] Arness MK, Eckart RE, Love SS, Altwood JE, Wells TS, Engler RJ et al Myopericarditis following smallpox vaccination. *Am J Epidemiol* 2004; 160: 642–51. doi: 10.1093/aje/kwh269 15383408 10.1093/aje/kwh269

[7] Halsell JS, Riddle JR, Atwood JE, Gardner P, Shope R, Poland GA et al Myopericarditis following smallpox vaccination among vaccinia-naive US military personnel. *JAMA* 2003; 289: 3283–89. doi: 10.1001/jama.289.24.3283 12824210 10.1001/jama.289.24.3283

[8] Sharma U, Tak T. A report of 2 cases of myopericarditis after Vaccinia virus (smallpox) immunization. *WMJ* 2011; 110: 291–94. 22324207

[9] Mora LF, Khan AH, Sperling LS. Cardiac complications after smallpox vaccination. *South Med J* 2009; 102: 615–19. doi: 10.1097/SMJ.0b013e31819fe55b 19434043 10.1097/SMJ.0b013e31819fe55b

[10] Suter M, Meisinger-Henschel C, Tzatzaris M, Huelsemann V, Lukassen S, Wulff NH. Modified Vaccinia Ankara strains with identical coding sequences actually represent complex mixtures of viruses that determine the biological properties of each strain. *Vaccine* 2009; 27: 7442–50. doi: 10.1016/j.vaccine.2009.05.095 19539582 10.1016/j.vaccine.2009.05.095

[11] Stittelaar KJ, van Amerongen G, Kondova I, Kuiken T, van Lavieren RF, Pistoor RH et al Modified vaccinia virus Ankara protects macaques against respiratory challenge with monkeypox virus. *J Virol* 2005; 79: 7845–51. doi: 10.1128/JVI.79.12.7845-7851.2005 15919938 10.1128/JVI.79.12.7845-7851.2005 PMC1143678

[12] Greenberg RN, Hay CM, Stapleton JT, Marbury TC, Wagner E, Kreitmeir E et al A Randomized, Double-Blind, Placebo-Controlled Phase II Trial Investigating the Safety and Immunogenicity of Modified Vaccinia Ankara Smallpox Vaccine (MVA-BN^®^) in 56-80-Year-Old Subjects. *PLoS One* 2016; 11: e0157335 doi: 10.1371/journal.pone.0157335 27327616 10.1371/journal.pone.0157335 PMC4915701

[13] Zitzmann-Roth EM, von Sonnenburg F, de la Motte S, Arndtz-Wiedermann N, von Krempelhuber A, Uebler N et al Cardiac Safety of Modified Vaccinia Ankara for Vaccination against Smallpox in a Young, Healthy Study Population. *PLoS One* 2015; 10: e0122653 doi: 10.1371/journal.pone.0122653 25879867 10.1371/journal.pone.0122653 PMC4399887

[14] Greenberg RN, Overton ET, Haas DW, Frank I, Goldman M, on Krempelhuber A et al Safety, Immunogenicity, and Surrogate Markers of Clinical Efficacy for Modified Vaccinia Ankara as a Smallpox Vaccine in HIV-Infected Subjects. *J Infect Dis* 2013; 207:749–58. doi: 10.1093/infdis/jis753 23225902 10.1093/infdis/jis753 PMC3611764

[15] Overton ET, Stapleton J, Frank I, Hassler S, Goepfert PA, Barker D et al Safety and Immunogenicity of Modified Vaccinia Ankara-Bavarian Nordic Smallpox Vaccine in Vaccinia-Naive and Experienced Human Immunodeficiency Virus-Infected Individuals: An Open-Label, Controlled Clinical Phase II Trial. *OFID* 2015; 2: doi: 10.1093/ofid/ofv040 26380340 10.1093/ofid/ofv040 PMC4567089

[16] Von Sonnenburg F, Perona P, Darsow U, Ring J, von Krempelhuber A, Vollmar J et al Safety and immunogenicity of modified vaccinia Ankara as a smallpox vaccine in people with atopic dermatitis. *Vaccine* 2014; 32: 5696–702. doi: 10.1016/j.vaccine.2014.08.022 25149431 10.1016/j.vaccine.2014.08.022

[17] Greenberg RN, Hurley Y, Dinh DV, Mraz S, Vera JG, von Bredow D et al A Multicenter, Open-Label, Controlled Phase II Study to Evaluate Safety and Immunogenicity of MVA Smallpox Vaccine (IMVAMUNE) in 18–40 Year Old Subjects with Diagnosed Atopic Dermatitis. *PLoS One* 2015; 10: e0138348 doi: 10.1371/journal.pone.0138348 26439129 10.1371/journal.pone.0138348 PMC4595076

[18] Frey SE, Newman FK, Kennedy JS, Sobek V, Ennis FA, Hill H et al Clinical and immunologic responses to multiple doses of IMVAMUNE^®^ (Modified Vaccinia Ankara) followed by Dryvax^®^ challenge. *Vaccine* 2007; 25: 8562–73. doi: 10.1016/j.vaccine.2007.10.017 18036708 10.1016/j.vaccine.2007.10.017 PMC2713577

[19] Frey SE, Winokur PL, Hill H, Goll JB, Chaplin P, Belshe RB. Phase II randomised, double-blinded comparison of a single high dose (5×10^8^ TCID_50_) of modified vaccinia Ankara compared to a standard dose (1×10^8^ TCID_50_) in healthy vaccinia-naïve individuals. *Vaccine* 2014; 32: 2732–39. doi: 10.1016/j.vaccine.2014.02.043 24607004 10.1016/j.vaccine.2014.02.043 PMC4106233

[20] Von Krempelhuber A, Vollmar J, Pokorny R, Rapp P, Wulff N, Petzold B et al A randomised, double-blind, dose-finding Phase II study to evaluate immunogenicity and safety of the third generation smallpox vaccine candidate IMVAMUNE^®^. Vaccine 2010; 28: 1209–16. doi: 10.1016/j.vaccine.2009.11.030 19944151 10.1016/j.vaccine.2009.11.030 PMC2814951

[21] Frey SE, Wald A, Edupuganti S, Jackson LA, Stapleton JT, El Sahly H et al Comparison of lyophilized versus liquid modified vaccinia Ankara (MVA) formulations and subcutaneous versus intradermal routes of administration in healthy vaccinia-naïve subjects. *Vaccine* 2015; 33: 5225–34. doi: 10.1016/j.vaccine.2015.06.075 26143613 10.1016/j.vaccine.2015.06.075 PMC9533873

[22] Vollmar J, Arndtz N, Eckl K, Thomsen T, Petzold B, Mateo L et al Safety and immunogenicity of IMVAMUNE, a promising candidate as a third generation smallpox vaccine. *Vaccine* 2006; 24: 2065–70. doi: 10.1016/j.vaccine.2005.11.022 16337719 10.1016/j.vaccine.2005.11.022

[23] Greenberg RN, Sheldon E, Willsie S, Bigger JE, Barnewall R, Gillum K et al Non-inferior immunogenicity and comparable safety and tolerability of a new freeze-dried compared to the current liquid frozen formulation of the non-replicating smallpox vaccine MVA-BN^®^. Manuscript in preparation.

[24] Elizaga M, Vasan S, Marovich M, Sato AH, Lawrence DN, Chaitman BR et al for the MVA Cardiac Safety Working Group. Prospective surveillance for cardiac adverse events in healthy adults receiving Modified Vaccinia Ankara vaccines: a systematic review. *PLoS One* 2013; 8: e54407 doi: 10.1371/journal.pone.0054407 23349878 10.1371/journal.pone.0054407 PMC3547923

[25] Haldiman L, Weimholt R, Singh G. Feasibility and usefulness of troponin I testing in insurance medicine. *J Insur Med* 2014; 44:184–88. 25622390

[26] Hingorani P, Natekar M, Deshmukh S, Karnad DR, Kothari S, Narula D et al Morphological abnormalities in baseline ECGs in healthy normal volunteers participating in phase I studies. *Indian J Med Res* 2012; 135: 322–30. 22561618 PMC3361868

[27] Rose G, Baxter PJ, Reid DD, McCartney P. Prevalence and prognosis of electrocardiographic findings in middle-aged men. *Br Heart J* 1978; 40: 636–43. 656238 10.1136/hrt.40.6.636 PMC483461

[28] Schwier N, Coons J, Rao S. Pharmacotherapy Update of Acute Idiopathic Pericarditis. *Pharmacotherapy* 2015; 35: doi: 10.1002/phar.1527 25630413 10.1002/phar.1527

[29] ACAM2000 Vaccines and Related Biological Products. Advisory Committee (VRBPAC) Briefing Document (April 2007) http://www.fda.gov/ohrms/dockets/ac/07/briefing/2007-4292B2-00-index.html (accessed September 1, 2016).

[30] Engler RJM, Nelson MR, Collins LC, Spooner C, Hemann BA, Gibbs BT et al A prospective study of the incidence of myocarditis/pericarditis and new onset cardiac symptoms following smallpox and influenza vaccination. *PLoS One* 2015; 10: e0118283 doi: 10.1371/journal.pone.0118283 25793705 10.1371/journal.pone.0118283 PMC4368609

