# Supplementary material for: Immunogenicity and safety of three consecutive production lots of the non replicating smallpox vaccine MVA: A randomised, double blind, placebo controlled phase III trial
Source: PLoS One. 2018 Apr 13;13(4):e0195897. doi: 10.1371/journal.pone.0195897 (PMC5898760; doi:10.1371/journal.pone.0195897)
Supplement: S1 File — (PDF) [file pone.0195897.s001.pdf]

A Clinical Phase III Trial Demonstrating Lot Consistency and Confirming Cardiac and Overall Safety of the Non-replicating Smallpox Vaccine MVA-BN®

Overton ET[1] for all POX-MVA-013 sites, Lawrence SJ[2], Wagner E[3], Nopora K[3], Rösch S[3], Young P[3], Schmidt D[3], Meyer TP[3], Weidenthaler H[3], Samy N[3], Chaplin P[3]

[1] University of Alabama at Birmingham School of Medicine, Birmingham, Alabama, USA
[2] Washington University School of Medicine, St. Louis, Missouri, USA
[3] Bavarian Nordic, Martinsried, Germany

Poster 144

Abstract

Background
Modified Vaccinia Ankara (MVA-BN®; invented name IMVANEX® within the EU, trade name IMVAMUNE® outside the EU) is a live, highly attenuated, non-replicating viral vaccine under advanced development as a non-replicating smallpox vaccine.

Methods
In this randomized, double-blind, placebo controlled, multicenter Phase III trial healthy, 18 to 40 year old vaccinia-naive subjects received two injections of either MVA-BN from one of three consecutively produced lots or placebo four weeks apart. To meet the objective of establishing lot consistency in regards to safety and immunogenicity, subjects were monitored for adverse events (AEs), particularly cardiac events. Immune responses were assessed using a plaque reduction neutralization test (PRNT) and an enzyme-linked immunosorbent assay (ELISA).

Results
4005 subjects received at least one injection (3003 MVA-BN, 1002 Placebo). Vaccination was well tolerated by all subjects without any safety concerns, in particular with regards to the development of cardiac diseases. A total of 25/3003 subjects (0.8%) vaccinated with MVA-BN reported 27 serious adverse events (SAEs). A total of 8/3003 subjects (0.3%) in the MVA-BN groups experienced an adverse event of special interest (AESI).

MVA-BN induced a strong immune response measured by PRNT and ELISA two weeks after the second vaccination, with seroconversion rates of 99.8% (PRNT) and 99.7% (ELISA). All MVA-BN groups were found to be equivalent per PRNT geometric mean titer (GMT) equivalence analysis.

Conclusions
In this large Phase III trial, MVA-BN elicited consistently robust humoral immunity across three separate production lots and demonstrated an excellent safety and tolerability profile comparable to previous trials, clearly differentiating MVA-BN from replicating smallpox vaccines.

Methods

This randomized, double-blind, placebo-controlled Phase III trial was conducted at 34 sites in the US (First subject screened: 18-Mar-2013. Last follow-up visit: 23-May-2014). A total of 4005 subjects were randomized into four groups. Healthy, vaccinia-naive women and men aged 18 to 40 years were eligible for enrolment. The trial consisted of a screening period of up to four weeks, an active trial period of eight to ten weeks comprised of five visits, and a follow-up (FU) period of 26 weeks after the second injection completed by a phone FU visit.

Vaccine Dose and Schedule
Group 1 to 3 received two subcutaneous (s.c.) injections with MVA-BN from three different consecutively produced lots at week 0 and week 4.
Group 4 received two s.c. injections with placebo (Tris-buffered saline) at week 0 and week 4.

Safety
To evaluate the safety of MVA-BN, solicited and unsolicited AEs were recorded at screening and at each of the five visits during the active trial phase. SAEs and AESIs were monitored throughout the trial up to the six months phone FU visit. In this trial an AESI is defined as any cardiac sign or symptom developed since the first vaccination, any clinically significant ECG change or Troponin I level ≥ 2 x ULN. In addition, safety laboratory tests including Troponin I, physical examinations including vital signs and electrocardiograms (ECG) were performed.

Criteria for evaluation:
- SAEs associated with the trial vaccine
- Any cardiac events and/or any ECG change indicating a case of myo-/pericarditis.
- Unsolicited non-serious AEs within 28 days after each vaccination
- Grade 3 or 4 AEs associated with the trial vaccine within 28 days after each vaccination.

Immunogenicity
Blood for serum collection was drawn at baseline prior to the first injection (week 0) and two weeks after the second injection (week 6). The humoral immune response was tested using vaccinia-specific ELISA and PRNT.

Criteria for evaluation of ELISA & PRNT:
- Percentage of seroconverting subjects
- Geometric mean titers (GMTs)

Statistical Methods:
The primary endpoint was tested using an equivalence analysis of vaccinia-specific neutralizing antibody GMTs induced by MVA-BN two weeks after the second vaccination based on the per protocol set (PPS). The trial was to demonstrate that the means of the PRNT $\log_{10}$ titers were equivalent within the margin of equivalence (Δ), predefined to be 0.301 for the $\log_{10}$ titers (this is equivalent to a factor of 2 for the GMT).
The primary analysis is presented in terms of the ratio of the GMTs (Group 1 / Group 2, Group 1 / Group 3 and Group 2 / Group 3) along with the 95% confidence interval (CI).

Acknowledgements: POX-MVA-013 was supported by Contract No.: HHSO100200700034C

Immunogenicity Results (Per Protocol Set)

PRNT GMT

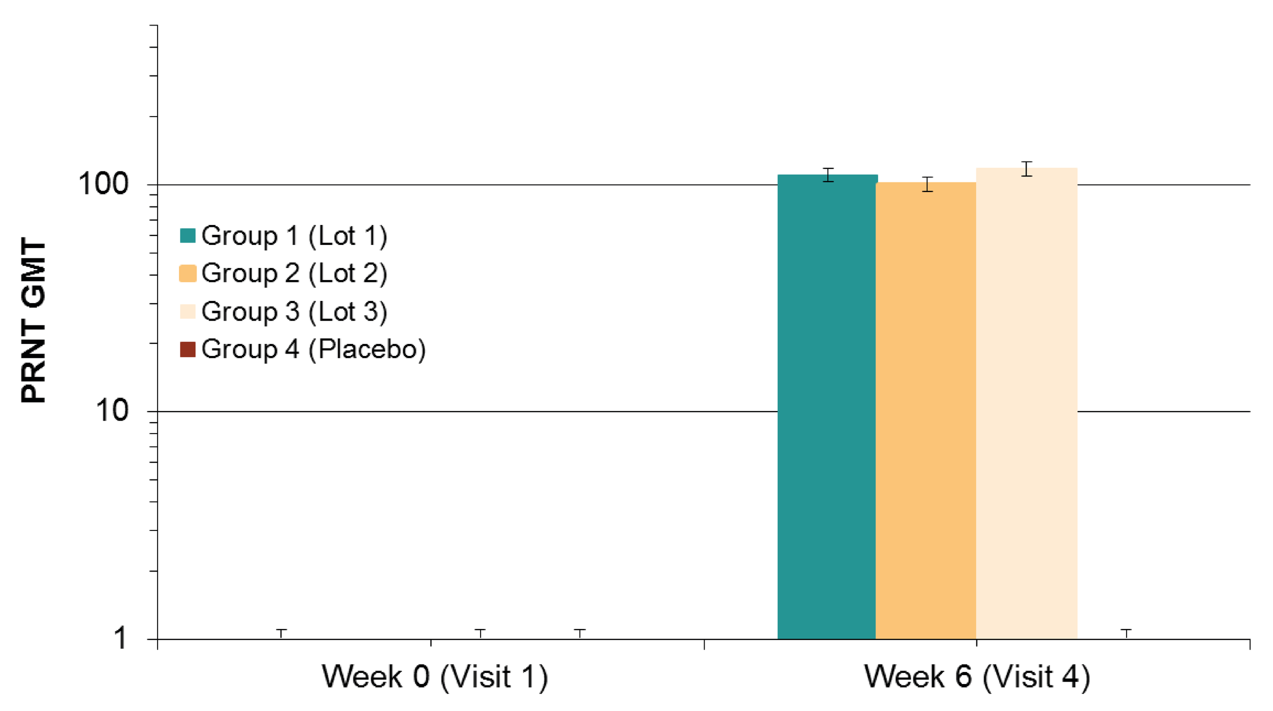

		Week 0 (Visit 1)	Week 6 (Visit 4)
Group 1 (Lot 1)	GMT	1.0	110.7
	CI	1.0, 1.1	103.4, 118.4
Group 2 (Lot 2)	GMT	1.0	100.5
	CI	1.0, 1.0	93.7, 107.8
Group 3 (Lot 3)	GMT	1.0	117.2
	CI	1.0, 1.1	109.0, 126.0
Group 4 (Placebo)	GMT	1.0	1.0
	CI	1.0, 1.1	1.0, 1.1

PRNT equivalence
Analysis of GMT post second injection

Group	GMT Ratio	95% CI [LCL, UCL]	Equivalence Met (Yes/No)
Group 1 / Group 2	1.1012	[0.9992, 1.2136]	Yes
Group 1 / Group 3	0.9444	[0.8554, 1.0427]	Yes
Group 2 / Group 3	0.8577	[0.7753, 0.9488]	Yes

All active treatment groups were equivalent thereby demonstrating lot consistency in terms of humoral immunogenicity.

ELISA GMT

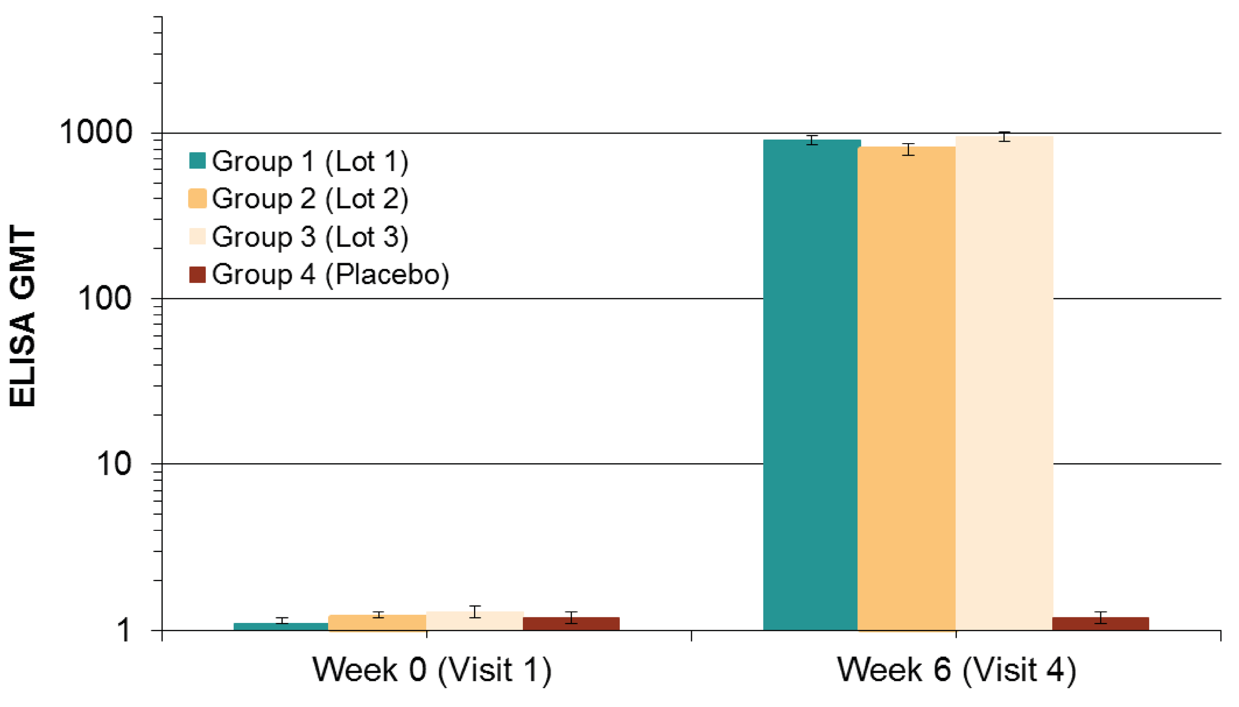

		Week 0 (Visit 1)	Week 6 (Visit 4)
Group 1 (Lot 1)	GMT	1.1	901.0
	CI	1.1, 1.2	844.8, 961.1
Group 2 (Lot 2)	GMT	1.2	794.4
	CI	1.2, 1.3	738.1, 855.0
Group 3 (Lot 3)	GMT	1.3	946.7
	CI	1.2, 1.4	888.2, 1008.9
Group 4 (Placebo)	GMT	1.2	1.2
	CI	1.1, 1.3	1.1, 1.3

Demographics (Full Analysis Set)

Group		Group 1 (N = 999)	Group 2 (N = 1005)	Group 3 (N = 999)	Group 1-3 (N = 3003)	Group 4 (N = 1002)
Age [years]	Mean	27.6	27.5	28.0	27.7	27.7
	SD	6.28	6.24	6.31	6.28	6.38
Gender n (%)	Male	473 (47.3)	478 (47.6)	505 (50.6)	1456 (48.5)	463 (46.2)
	Female	526 (52.7)	527 (52.4)	494 (49.4)	1547 (51.5)	539 (53.8)
Race n (%)	White/ Caucasian	773 (77.4)	790 (78.6)	765 (76.6)	2328 (77.5)	773 (77.1)
	Black/ African American	172 (17.2)	165 (16.4)	191 (19.1)	528 (17.6)	184 (18.4)
	Oriental/ Asian	24 (2.4)	17 (1.7)	18 (1.8)	59 (2.0)	19 (1.9)
	Other	30 (3.0)	33 (3.3)	25 (2.5)	88 (2.9)	26 (2.6)

There were no statistically significant differences for any demographic characteristics among treatment groups.

Safety Results (FAS)

Summary of Adverse Events

	Group 1 (N = 999)	Group 2 (N = 1005)	Group 3 (N = 999)	Group 1-3 (N = 3003)	Group 4 (N = 1002)
SAE	11 (1.1)*	7 (0.7)	7 (0.7)	25 (0.8)	8 (0.8)
At least possibly related SAE	0 (0.0)	0 (0.0)	0 (0.0)	0 (0.0)	1 (0.1)
AESI	2 (0.2)	5 (0.5)	1 (0.1)	8 (0.3)	1 (0.1)
At least possibly related AESI	0 (0.0)	1 (0.1)	1 (0.1)	2 (0.1)	0 (0.0)
Unsolicited AE	199 (19.9)	238 (23.7)	223 (22.3)	660 (22.0)	189 (18.9)
At least possibly related unsolicited AE	54 (5.4)	59 (5.9)	67 (6.7)	180 (6.0)	29 (2.9)
At least possibly related unsolicited AE Grade ≥ 3	2 (0.2)	2 (0.2)	2 (0.2)	6 (0.2)	1 (0.1)
AE leading to withdrawal from trial	5 (0.5)	9 (0.9)	4 (0.4)	18 (0.6)	3 (0.3)

* One subject in Group 1 died during the clinical trial. The subject committed suicide 19 days after receiving the first injection. The investigator assessed this death as treatment unrelated.

Adverse Events of Special Interest

	Preferred term	Relationship to trial vaccine	Outcome
Group 1 (Lot 1)	Troponin I increase ≥ 2 x ULN	Unlikely	Recovered
	Wolff-Parkinson-White Syndrome	Unrelated	Recovered
Group 2 (Lot 2)	Troponin I increase ≥ 2 x ULN	Unlikely	Recovered
	Supraventricular extra systoles	Unrelated	Recovered
	Bundle branch block right	Possible	Not Recovered
	Tachycardia	Unrelated	Recovered
	ECG ST Segment abnormal	Unrelated	Unknown
Group 3 (Lot 3)	Pericarditis*	Possible	Recovered
Group 4 (Placebo)	Bundle branch block right	Unrelated	Recovered

* A single case of chest pain, described as being worse when lying down and therefore meeting the protocol criterion of "possible acute pericarditis". The diagnosis was purely based on the clinical observation (chest pain being worse when lying down), while no abnormalities could be confirmed by a thorough cardiac examination of this subject. In summary, it remains doubtful if this case can be considered a case of pericarditis.

Conclusions

The primary objective of the trial was met: consistency of three consecutively produced MVA-BN lots was proven in terms of humoral immunogenicity (PRNT GMT equivalence).

No case of myocarditis and no confirmed case of pericarditis in more than 7000 subjects across all completed trials demonstrating the superior cardiac safety profile of MVA-BN as compared to replicating smallpox vaccines.

No safety concerns in this healthy, vaccinia-naive trial population and no differences in the safety profile across the three MVA-BN groups were observed.
