# Supplementary material for: Immunogenicity and safety of three consecutive production lots of the non replicating smallpox vaccine MVA: A randomised, double blind, placebo controlled phase III trial
Source: PLoS One. 2018 Apr 13;13(4):e0195897. doi: 10.1371/journal.pone.0195897 (PMC5898760; doi:10.1371/journal.pone.0195897)
Supplement: S3 File — (PDF) [file pone.0195897.s003.pdf]

POX-MVA-013

A Randomized, Double-Blind, Placebo-Controlled Phase III Trial to Evaluate Immunogenicity and Safety of Three Consecutive Production Lots of IMVAMUNE® (MVA-BN®) Smallpox Vaccine in Healthy, Vaccinia-Naïve Subjects

Final Clinical Trial Protocol Edition 2.0

1 General Information

1.1 Investigator Signature Page

Herewith I agree that I have read and fully understand this protocol:

A randomized, double-blind, placebo-controlled Phase III trial to evaluate immunogenicity and safety of three consecutive production lots of IMVAMUNE® (MVA-BN®) smallpox vaccine in healthy, vaccinia-naive subjects, Edition 2.0 dated 14-Aug-2013.

This protocol describes the information necessary to conduct the trial. I agree that I will conduct the trial according to the instructions given within this protocol. Furthermore, I agree that I will conduct this trial according to International Conference of Harmonization of Technical Requirements for Registration of Pharmaceuticals for Human Use (ICH) Good Clinical Practice (GCP), the 1996 version of the Declaration of Helsinki, as well as applicable local legal and regulatory requirements in the respective countries. I agree that all information revealed in this protocol is handled strictly confidentially.

Additionally, I will permit trial related monitoring, audits, Institutional Review Board (IRB) / Independent Ethics Committee (IEC) review and regulatory inspections, providing direct access to source data/documents.

____________________ Date	____________________ [name] Principal Investigator (PI), [clinical trial site (CTS)]

____

____

____
[CTS address]

1.2 Coordinating Investigator Signature Page

By signing the protocol:

A randomized, double-blind, placebo-controlled Phase III trial to evaluate immunogenicity and safety of three consecutive production lots of IMVAMUNE® (MVA-BN®) smallpox vaccine in healthy, vaccinia-naive subjects, Edition 2.0 dated 14-Aug-2013.

I agree, that the protocol was written according to international ethical and scientific quality standards (ICH-GCP), in compliance with the 1996 version of the Declaration of Helsinki and applicable local legal and regulatory requirements in the respective countries.

16-Aug-2013
Date

Edgar Turner Overton
Edgar Turner Overton, M.D.
Coordinating Investigator

Division of Infectious Diseases
University of Alabama at Birmingham
908 20th Street South
CCB Rm 325
Birmingham, AL 35294, USA

1.3 Sponsor Signature Page

By signing the protocol:

A randomized, double-blind, placebo-controlled Phase III trial to evaluate immunogenicity and safety of three consecutive production lots of IMVAMUNE® (MVA-BN®) smallpox vaccine in healthy, vaccinia-naive subjects, Edition 2.0 dated 14-Aug-2013.

The undersigned parties agree, that the protocol was written according to international ethical and scientific quality standards (ICH-GCP), in compliance with the 1996 version of the Declaration of Helsinki and applicable local legal and regulatory requirements in the respective countries.

16-Aug-2013
Date

Dr. Eva Wagner
Coordinating Author, Bavarian Nordic GmbH

16-Aug-2013
Date

Sanja Vidojkovic, MD
Medical Monitor, Bavarian Nordic GmbH

16-Aug-2013
Date

Dr. Thomas Meyer
Manager Clinical Analysis, Bavarian Nordic GmbH

16-Aug-2013
Date

Philip Young, PhD
Biostatistician, Bavarian Nordic GmbH

16-Aug-2013
Date

Monika Wodzien
QA Auditor GCP, Bavarian Nordic GmbH

16-AUG-2013
Date

Dr. Nathaly Arndtz-Wiedemann
Vice President Medical Affairs
Bavarian Nordic GmbH

1.4 Responsibilities

Trial Number	POX-MVA-013
Title	A randomized, double-blind, placebo-controlled Phase III trial to evaluate immunogenicity and safety of three consecutive production lots of IMVAMUNE® (MVA-BN®) smallpox vaccine in healthy, vaccinia-naïve subjects
Coordinating Investigator	Edgar Turner Overton, M.D.<br>Division of Infectious Diseases<br>University of Alabama at Birmingham<br>908 20th Street South<br>CCB Rm 325<br>Birmingham, AL 35294<br>USA
Phone	+1 205 934-5191
Fax	+1 205 975-6027
E-mail	toverton@uab.edu
Sponsor and Product Supply IMVAMUNE®	Bavarian Nordic A/S<br>Hejreskovvej 10A<br>DK-3490 Kvistgård, Denmark
Phone	+45 33 268 383
Fax	+45 33 268 380
Product Manufacturer (Placebo)	Bavarian Nordic GmbH<br>Robert-Roessle-Strasse 10<br>13125 Berlin, Germany
Project Leader	Dr. Eva Wagner<br>Bavarian Nordic GmbH<br>Fraunhoferstrasse 13<br>82152 Martinsried, Germany
Phone	+49 89 255 446 342
Fax	+49 89 255 446 333
E-mail	eva.wagner@bavarian-nordic.com
Medical Monitor	Sanja Vidojkovic, MD<br><br>Bavarian Nordic GmbH<br>Fraunhoferstrasse 13<br>82152 Martinsried, Germany
Phone	+49 89 255 446 479
Fax	+49 89 255 446 419
E-mail	sanja.vidojkovic@bavarian-nordic.com

Trial Statistician	Philip Young, PhD<br>Bavarian Nordic GmbH<br>Fraunhoferstrasse 13<br>82152 Martinsried, Germany
Phone	+49 89 255 446 462
Fax	+49 89 255 446 333
E-mail	philip.young@bavarian-nordic.com
Chiltern/Project Manager	Doug Clendenon<br>Chiltern International Ltd<br>1241 Volunteer Parkway<br>Suite 950<br>Bristol, TN 37620, USA
Phone	+1 423 990 0466
Fax	+1 423 968 3567
E-mail	doug.clendenon@chiltern.com
Laboratory (Safety)/<br>Project Manager	Brian Bissell<br>Quintiles Laboratories, Ltd.<br>1600 Terrell Mill Road, Ste. 100<br>Marietta, GA 30067, USA
Phone	+1 404 434 9061
Fax	+1 404 890 5450
E-mail	brian.bissell@quintiles.com
Laboratory (Immunology)	Dr. Thomas Meyer<br>Bavarian Nordic GmbH<br>Fraunhoferstrasse 13<br>82152 Martinsried, Germany
Phone	+49 89 255 446 405
Fax	+49 89 255 446 333
E-mail	thomas.meyer@bavarian-nordic.com
Centralized ECG Assessment<br>Project Manager	Kailyn Bell<br>Quintiles Cardiac Services
Phone	+1 267 886 8477
Fax	+1 215 933 3221
E-mail	kailyn.bell@quintiles.com

Table of Contents

1 General Information 2
1.1 Investigator Signature Page 2
1.2 Coordinating Investigator Signature Page 3
1.3 Sponsor Signature Page 4
1.4 Responsibilities 5
1.5 Protocol Synopsis 13
1.6 Trial Procedure Schedule 19
2 Background Information and Scientific Rationale 20
2.1 Introduction 20
2.2 First Generation Smallpox Vaccines 20
2.3 Second Generation Smallpox Vaccines 21
2.4 Origin and Characteristics of IMVAMUNE® 22
2.5 Summary of Preclinical Data with IMVAMUNE® 22
2.6 Clinical Profile of IMVAMUNE® 23
2.6.1 Safety Overview of IMVAMUNE® 23
2.6.2 Immunogenicity Overview of IMVAMUNE® 26
2.7 Rationale 27
2.8 Trial Population 27
2.9 Risk/Benefit Assessment 28
2.9.1 Potential Risks 28
2.9.2 Benefits 28
3 Objectives 28
4 Trial Design 29
4.1 Experimental Design 29
4.2 Description of Trial Procedures 29
4.2.1 Screening Phase 29

4.2.2 Active Trial Phase 30
4.2.3 Follow-Up (FU) Phase 33
4.2.4 Unscheduled Visits 34
4.2.5 Withdrawal from Second Vaccination 34
4.2.6 Premature Discontinuation 34
4.2.7 Emergency Unblinding 35
4.3 Trial Duration 35
4.4 Data Safety Monitoring Board 35
4.5 Trial Halting Rules 36
5 Selection of Subjects 36
5.1 Recruitment Procedure 36
5.2 Inclusion Criteria 36
5.3 Exclusion Criteria 36
6 Investigational Product 37
6.1 Production, Packaging and Labeling 37
6.2 Shipment, Storage and Handling 37
6.3 Preparation, Administration and Dosage 38
6.4 Accountability and Disposal 38
7 Assessment of Immunogenicity 39
7.1 Humoral Immunogenicity 39
7.2 Future Use of Lab Specimen 40
8 Safety and Reactogenicity 40
8.1 Definitions 40
8.1.1 Medical History 40
8.1.2 Baseline Signs and Symptoms 40
8.1.3 AE 40
8.2 Assessment 42
8.2.1 Relevant Medical History 42
8.2.2 Prior and Concomitant Medications 42

8.2.3 Physical Examination and Vital Signs 42
8.2.4 Vital signs 43
8.2.5 Unsolicited AE 43
8.2.6 Solicited AE 44
8.2.7 Cardiac Assessment 46
8.2.8 Safety Laboratory Measurements 49
8.2.9 Pregnancy 49
8.3 Reporting 50
8.3.1 Reporting of SAE 50
8.3.2 Reporting of AESI 52
8.3.3 Reporting of Pregnancies 54
9 Statistical Considerations 54
9.1 Primary Trial Hypothesis 54
9.2 Endpoints 55
9.3 Sample Size Calculation 55
9.4 Trial Cohorts/Datasets to be Evaluated 56
9.5 Biometrical Evaluation 57
10 Ethical Aspects 57
10.1 Ethical and Legal Regulations 57
10.2 Approval by an IEC / IRB 57
10.3 Confidentiality and Data Protection 58
11 Informed Consent 58
12 eCRFs and Retention of Records 59
12.1 eCRF 59
12.2 Retention of Records 59
13 Monitoring of the Trial 59
14 Audits and Inspections 60
15 Responsibilities of the Investigator 60
16 References 61

17 Appendices ... 63
17.1 Appendix I: Toxicity Scale for Laboratory Values ... 63
17.2 Appendix II: Case Definitions Acute Myocarditis / Pericarditis ... 64
17.3 Appendix III: Interpretation Support for Assessment of Screening ECGs ... 67
17.4 Appendix IV: Grading Scale for Lymphadenopathy ... 68
17.5 Appendix V: Amendment #1 ... 69
17.5.1 Rationale ... 70
17.5.2 Changes ... 70

Table of Tables

Table 1: Serious Suspected ADRs (SAEs Assessed by the Investigator to Be At Least Possibly Related to IMVAMUNE®) ... 25
Table 2: Grading of General Symptoms from the Subject's Memory Aid ... 46
Table 3: Toxicity Scale for Serum Chemistry ... 64
Table 4: Toxicity Scale for Hematology ... 64

Tables of Figures

Figure 1: Algorithm for Assessment of Cardiac Events ... 48
Figure 2: Algorithm for Reporting of SAEs ... 51
Figure 3: Algorithm for Reporting of AESIs ... 53

List of Abbreviations

AD	Atopic Dermatitis
ADR	Adverse Drug Reaction
AE	Adverse Event
AESI	Adverse Event of Special Interest
ALT	Alanine Aminotransferase
ANC	Absolute Neutrophil Count
AST	Aspartate Aminotransferase
BARDA	Biomedical Advanced Research and Development Authority
BN	Bavarian Nordic
CDISC	Clinical Data Interchange Standards Consortium
CrCL	Creatinine Clearance
CRA	Clinical Research Associate
CRO	Contract Research Organization
CTS	Clinical Trial Site
CVA	Chorioallantois Vaccinia Virus Ankara
DMID	Division of Microbiology and Infectious Diseases
DS	Drug Safety
DSMB	Data Safety Monitoring Board
ECG	Electrocardiogram
eCRF(s)	Electronic Case Report Form(s)
ELISA	Enzyme-linked Immunosorbent Assay
FAS	Full Analysis Set
FDA	United States Food and Drug Administration
FU	Follow-up
GCP	Good Clinical Practice
GH-RH	Growth Hormone Releasing Hormone
GMP	Good Manufacturing Practice
GMT	Geometric Mean Titer
HBV	Hepatitis-B-Virus
HCG	Human Choriogonadotropin
HCV	Hepatitis-C-Virus
HIPAA	Health Insurance Portability and Accountability Act
HIV	Human Immunodeficiency Virus
ICF	Informed Consent Form
ICH	International Conference of Harmonization of Technical Requirements for Registration of Pharmaceuticals for Human Use
IEC	Independent Ethical Committee
IVRS	Interactive Voice Response System
IWRS	Interactive Web Response System
i.m.	Intramuscular
IND	Investigational New Drug (Application)
IMP	Investigational Medicinal Product

IRB	Institutional Review Board
LLN	Lower Limit of Normal
LV	Left Ventricular
MedDRA	Medical Dictionary for Regulatory Activities
MP	Medical Product
MVA	Modified Vaccinia Ankara Strain
MVA-BN®	Modified Vaccinia Ankara – Bavarian Nordic
NHP	Non-Human Primates
NIAID	National Institute of Allergy and Infectious Diseases
NIH	National Institutes of Health
NYCBH	New York City Board of Health
ODM	Operational Data Modeling
PEI	Paul Ehrlich Institut
PI	Principal Investigator
PPS	Per Protocol Set
PRNT	Plaque Reduction Neutralization Test
SADR	Serious Adverse Drug Reaction
SAE	Serious Adverse Event
s.c.	Subcutaneous
SCR	Screening Visit
SD	Standard Deviation
TBS	Tris-buffered Saline
$TCID_{50}$	Tissue Culture Infectious Dose 50%
ULN	Upper Limit of Normal
US(A)	United States (of America)
V	Visit
VRBPAC	Vaccines and Related Biological Products Advisory Committee
VV	Vaccinia Virus
VV-WR	Vaccinia Virus Western Reserve
WBC	White Blood Cell Count
WHO	World Health Organization
WOCBP	Women of Childbearing Potential

1.5 Protocol Synopsis

Title	A randomized, double-blind, placebo-controlled Phase III trial to evaluate immunogenicity and safety of three consecutive production lots of IMVAMUNE® (MVA-BN®) smallpox vaccine in healthy, vaccinia-naïve subjects.
Clinical phase	Phase III
Sponsor	Bavarian Nordic A/S<br>Hejreskovvej 10A<br>DK-3490 Kvistgård, Denmark
Coordinating Investigator	Edgar Turner Overton, M.D.<br>Division of Infectious Diseases<br>University of Alabama at Birmingham<br>908 20th Street South<br>CCB Rm 325<br>Birmingham, AL 35294, USA
Number of clinical trial sites and country/ies	Up to 40 in the United States of America (USA)
Vaccination dose and schedule	Two vaccinations four weeks apart (at Day 0 and Day 28) with either 0.5 ml IMVAMUNE® vaccine containing a nominal titer of 1×10^8 $TCID_{50}$ (standard dose) or 0.5 ml placebo (Tris-buffered saline, TBS).
Route of administration	Each IMVAMUNE® vaccination / placebo administration consists of one subcutaneous (s.c.) injection.
Trial duration	Up to 39 weeks for each subject.
Sample size	4,000 (1,000 subjects per group).

Primary objective	To assess the consistency of three consecutively produced IMVAMUNE® lots.
Secondary objectives	To assess uncommon adverse reactions, in particular any cardiac sign and symptom indicating a case of myo-/pericarditis, and to compare the frequency of those reactions against placebo.<br><br>To collect vaccinia-specific humoral immune response data.
Primary endpoint	Geometric Mean Titers (GMTs) after two IMVAMUNE® vaccinations measured by Plaque Reduction Neutralization Test (PRNT) at trial Visit 4.
Secondary endpoints	**Immunogenicity**<br><br>GMTs after two IMVAMUNE® vaccinations measured by Enzyme-linked Immunosorbent Assay (ELISA) at trial Visit 4.<br><br>PRNT and ELISA seroconversion rates at trial Visit 4.<br><br>Pearson Correlation Coefficient between the log10 transformed PRNT titers and the log10 transformed ELISA titers at trial Visit 4.<br><br>**Safety and Reactogenicity**<br><br>Occurrence, relationship and intensity of any Serious Adverse Event (SAE) at any time during the trial.<br><br>Occurrence, relationship and intensity of any cardiac sign or symptom indicating a case of myo-/pericarditis.<br><br>Occurrence of any Grade 3 or 4 adverse events (AEs) probably, possibly or definitely related to the trial vaccine within 28 days after vaccination.<br><br>Occurrence, relationship and intensity of unsolicited non-serious AEs within 28 days after each vaccination.<br><br>Occurrence, intensity and duration of solicited local AEs (redness, swelling, induration, pruritus and pain) during the 8-day period (day of vaccination and the following 7 days) after each vaccination.<br><br>Occurrence, relationship, intensity and duration of solicited general

AEs (pyrexia, headache, myalgia, nausea, fatigue and chills) during the 8-day period (day of vaccination and the following 7 days) after each vaccination.

Trial design

Randomized, double-blind, placebo-controlled:

Group 1	1,000 subjects will receive two s.c. vaccinations with 0.5 ml IMVAMUNE® Lot 1
Group 2	1,000 subjects will receive two s.c. vaccinations with 0.5 ml IMVAMUNE® Lot 2
Group 3	1,000 subjects will receive two s.c. vaccinations with 0.5 ml IMVAMUNE® Lot 3
Group 4	1,000 subjects will receive two s.c. vaccinations with 0.5 ml Placebo (TBS)

Visit Schedule:

**Visit (V)**	**Day**	**Target Week**	**Vaccination**
**SCR**	Day -28 to -1	-4	
**V1**	Day 0	0	X
**V2**	V1 +12 - 16	2	
**V3**	V1 +28 - 35	4	X
**V4**	V3 +12 - 16	6	
**V5**	V3 +28 - 35	8	
**Phone FU**	V3 +182 - 210	30	

Subject entry criteria

Inclusion criteria

1. Male and female subjects, 18 to 40 years of age
2. The subject has read, signed and dated the informed consent form, having been advised of the risks and benefits of the trial in a language understood by the subject and prior to performance of any trial specific procedures
3. BMI ≥ 18.5 and < 35
4. Women of childbearing potential (WOCBP) must have used an acceptable method of contraception for 30 days prior to the first vaccination, must agree to use an acceptable method of contraception during the trial, and must avoid becoming pregnant for at least 28 days after the last vaccination. A woman is considered of childbearing potential unless post-menopausal or

with a history of hysterectomy (Acceptable contraception methods are restricted to abstinence, barrier contraceptives, intrauterine contraceptive devices or licensed hormonal products)

5. WOCBP must have a negative serum pregnancy test at screening and a negative urine pregnancy test within 24 hours prior to each vaccination
6. White blood cells $\geq 2{,}500/mm^3 <$ ULN
7. Absolute neutrophil count (ANC) within normal limits
8. Hemoglobin within normal limits
9. Platelets within normal limits
10. Adequate renal function defined as a calculated Creatinine Clearance (CrCl) > 60 ml/min as estimated by the Cockcroft-Gault equation:
    - For men: (140 – age in years) x (body weight in kg) ÷ (serum creatinine in mg/dl x 72) = CrCl (ml/min)
    - For women: multiply the result by 0.85 = CrCl (ml/min).
11. Adequate hepatic function defined as:
    - a. Total bilirubin ≤ 1.5 x ULN in the absence of other evidence of significant liver disease
    - b. Aspartate aminotransferase (AST), alanine aminotransferase (ALT) and alkaline phosphatase ≤ 1.5 x ULN
12. Troponin I < 2 x ULN
13. Electrocardiogram (ECG) without clinically significant findings (e.g. any kind of atrioventricular or intraventricular conditions or blocks such as complete left or right bundle branch block, AV node block, QTc or PR prolongation, premature atrial contractions or other atrial arrhythmia, sustained ventricular arrhythmia, two premature ventricular contractions in a row, ST elevation consistent with ischemia)

Exclusion criteria

1. Typical vaccinia scar
2. Known or suspected history of smallpox vaccination
3. History of vaccination with any poxvirus-based vaccine
4. US Military service prior to 1991 or after January 2003
5. Pregnant or breast-feeding women

6. Uncontrolled serious infection, i.e. not responding to antimicrobial therapy
7. History of any serious medical condition, which in the opinion of the investigator would compromise the safety of the subject or would limit the subject's ability to complete the trial
8. History of or active autoimmune disease, persons with vitiligo or thyroid disease taking thyroid replacement are not excluded
9. Known or suspected impairment of immunologic function including, but not limited to, HIV Infection, clinically significant liver disease (including chronic active HBV or HCV), diabetes mellitus, moderate to severe kidney impairment
10. History of malignancy other than squamous cell or basal cell skin cancer, unless there has been surgical excision that is considered to have achieved cure. Subjects with history of skin cancer must not be vaccinated at the previous tumor site
11. History or clinical manifestation of clinically significant and severe hematological, pulmonary, central nervous, cardiovascular or gastrointestinal disorders
12. Clinically significant mental disorder not adequately controlled by medical treatment
13. History of coronary heart disease, myocardial infarction, angina pectoris, congestive heart failure, cardiomyopathy, stroke or transient ischemic attack, uncontrolled high blood pressure, or any other heart condition under the care of a doctor
14. Known history of an immediate family member (father, mother, brother, or sister) who has had onset of ischemic heart disease before age 50 years
15. Ten percent or greater risk of developing a myocardial infarction or coronary death within the next 10 years using the National Cholesterol Education Program's risk assessment tool (http://hin.nhlbi.nih.gov/atpiii/calculator.asp?usertype=prof) NOTE: This criterion applies only to subjects 20 years of age and older
16. Active or history of chronic alcohol abuse and/or intravenous and/or nasal drug abuse (within the past 6 months)
17. Known allergy to IMVAMUNE® vaccine and its constituents, e.g. tris (hydroxymethyl)-amino methane, including know allergy to egg or aminoglycosides
18. History of anaphylaxis or severe allergic reaction to any vaccine
19. Acute disease (illness with or without a fever) at the time

enrollment

20. Body temperature ≥100.4°F (≥38.0°C) at the time of enrollment
21. Having received any vaccinations or planned vaccinations with a live vaccine within 30 days prior to or after trial vaccination
22. Having received any vaccinations or planned vaccinations with a killed vaccine within 14 days prior to or after trial vaccination
23. Chronic systemic administration (defined as more than 14 days) of > 5 mg prednisone (or equivalent)/day or any other immune-modifying drugs during a period starting from three months prior to administration of the vaccine and ending at last physical trial visit (V5)
24. Post organ transplant subjects whether or not receiving chronic immunosuppressive therapy
25. Administration or planned administration of immunoglobulins and/or any blood products during a period starting from three months prior to administration of the vaccine and ending at last physical trial visit (V5)
26. Use of any investigational or non-registered drug or vaccine other than the trial vaccine within 30 days preceding the first dose of the trial vaccine, or planned administration of such a drug during the trial period
27. Trial personnel

1.6 Trial Procedure Schedule

Visit (V)	SCR	V1	V2	V3	V4	V5	FU Phone
Day / Visit +… D	-28- -1	0	V1 +12-16	V1 +28-35	V3 +12-16	V3 +28-35	V3 +182-210
Target week	- 4	0	2	4	6	8	30
**Procedures**							
Informed consent	X						
Check incl. / excl. criteria	X	X					
Check withdrawal criteria				X			
Medical History	X						
Assessment for previous smallpox vaccination including check for a scar	X						
Complete physical exam	X						
Evaluation of vital signs	X	X	X	X	X	X	(X)[1]
Calculate individual cardiac risk factor	X						
Evaluation of family cardiac risk factors	X						
Recording of baseline signs and symptoms	X	X					
Targeted physical exam incl. auscultation of the heart and lung		X	X	X	X	X	(X)[1]
ECG[5]	X		X		(X)[2]		
Recording of prior and concomitant medication	X	X	X	X	X	X	
Counseling on avoidance of pregnancy for WOCBP[7]	X	X		X			
AE/SAE/AESI recording		X	X	X	X	X	X[3]
**Lab**							
Pregnancy test for WOCBP[4]	X	X		X		X	
Obtaining blood for safety lab[5]	X		X		X		(X)[1]
Total, HDL and LDL cholesterol	X						
Troponin I testing[5]	X		X		(X)[2]		
Sera collection for antibody analysis		X			X		
**Vaccination**							
Vaccine administration & Subject observation (≥ 30 minutes)		X		X			
Recording of immediate AEs		X		X			
Handout of memory aid		X		X			
Collection of memory aid			X		X		
Examination of injection site			X		X		
**Blood volume**							
Appr. blood volume drawn (ml)[5, 6]	11	8	11	0	19	0	(11)[1]
Cumulative blood volume drawn (ml)[5]	11	19	30	30	49	49	(60)[1]

1 If at the telephone FU a serious condition is detected, the trial subject will be requested to return to the CTS and the respective examinations will be performed.
2 Only if clinically indicated, i.e. in the presence of cardiac signs or symptoms.
3 New SAEs/AESIs and changes to SAEs/AESIs/AEs ongoing at V5 only.
4 At SCR, a serum test must be performed. At other visits, a urine pregnancy test will be performed.
5 If clinically indicated, additional safety measures can be taken at any other trial visits or at unscheduled visits.
6 Approximate amounts of single blood draws: Safety lab including all tests: 11 ml; antibody analysis: 8 ml.
7 Review of acceptable contraceptive methods and recent menstrual history with WOCBP.
(x) Only to be performed if clinically indicated.

2 Background Information and Scientific Rationale

2.1 Introduction

Despite the fact that the World Health Organization (WHO) officially declared successful global eradication of smallpox in 1980, the existence of variola stockpiles and the threat of bioterrorism demands to maintain immunity to smallpox through vaccination. After the events of September 11th, 2001, concern over the use of bioweapons as agents of terrorism increased (McCurdy et al. 2004). As mass vaccination programs halted more than 30 years ago, it is estimated that the majority of the world population has no existing immunity to smallpox, and as such, the release of this highly contagious virus would have devastating effects. As a consequence, there is an urgent need for a safe and efficacious vaccine to protect the public against smallpox.

2.2 First Generation Smallpox Vaccines

The original smallpox vaccines were based on a number of different vaccinia virus (VV) strains, e.g. Lister-Elstree strain recommended by the WHO and used primarily in Europe or the New York City Board of Health (NYCBH, Dryvax®) strain used in the United States. While these proved to be highly effective immunizing agents making the eradication of smallpox possible, they also showed considerable side effects. Besides local reactions with scab development and scarring, general symptoms observed frequently after smallpox vaccination have been pyrexia, weakness, muscular pain, headache, swelling and soreness of local lymph nodes and rashes. Pyrexia occurred in the majority of vaccinees, especially in small children. Apart from less dramatic and transient side effects like erythematous or urticarial rashes, severe and potentially fatal cutaneous complications of vaccinia vaccination include eczema vaccinatum and progressive vaccinia. Most feared are complications of the central nervous system, especially post-vaccinal encephalitis, which lead to death in 15-25% of cases and in 25% to neurologic sequelae (Goldstein et al. 1975; Lane et al. 1969; Lane et al. 1970). In Germany, the occurrence of neurological complications in primary vaccinees was reported in 1:20,000 to 30,000 vaccinees. Even though some countries such as the United States excluded high-risk individuals from vaccination, an average of seven persons a year still died from complications due to smallpox vaccination during the eradication campaign (McElwain 1972).

Replication competent smallpox vaccines could be lethal if given to immune compromised individuals and are therefore contraindicated for e.g. persons who have received organ transplants, persons with cancer, Atopic Dermatitis (AD) or HIV. A trial published in 1991 (Guillaume et al. 1991) reported two cases of HIV infected immune compromised patients who experienced necrotic skin lesions due to generalized vaccinia infections that led to death. However, complications following vaccinations with vaccinia can also occur in HIV infected individuals with T cell counts in the normal range and who are otherwise healthy (Redfield et al. 1987).

Traditionally, successful vaccination with a smallpox vaccine was assessed based on the formation of a vesicle ("take") at the inoculation site seven to nine days after vaccination. Recent clinical trials using Dryvax® confirmed a success rate by vesicle formation in vaccinia-naïve

subjects of 95 - 99% (Frey et al, 2002; ACAM2000 Vaccines and Related Biological Products Advisory Committee [VRBPAC] Briefing Document, 2007).

2.3 Second Generation Smallpox Vaccines

Second generation smallpox vaccines are derived from first generation VV strains by plaque purification and manufactured in cell cultures according to modern Good Manufacturing Practice (GMP) standards. Vaccination of individuals with these vaccines is performed in the same way as with first generation smallpox vaccines, namely by intradermal administration (scarification) of a single dose.

ACAM2000® developed by Acambis Inc. is based on the Dryvax® NYCBH strain. In preparation of a Biologics License Application at the US Food and Drug Administration (FDA), Acambis conducted two pivotal Phase III clinical trials enrolling either vaccinia-naïve or vaccinia-experienced populations. The trials were designed to compare the safety, tolerability and efficacy of ACAM2000® with Dryvax®. In total, the ratio of individuals in these trials receiving ACAM2000® and Dryvax® was 3:1. Results were publicly made available in May 2007 (ACAM2000 VRBPAC Briefing Document, 2007).

Safety information available from these trials suggests that the non-serious adverse reactions were typical for vaccines administered by injection or scarification. The majority (99% and 97% respectively) of subjects experienced at least one treatment-emergent AE after vaccination. The AEs most commonly reported fell into four distinct categories: reactions at the vaccination site, lymphadenitis, constitutional "flu-like" symptoms and minor gastrointestinal symptoms. Of special interest, however, were a total of 10 serious cases of myo-/pericarditis that were reported within the ACAM2000® development program. In a total vaccinia-naïve population of 1,675 subjects, these events occurred in seven subjects treated with ACAM2000® (5.73 events per thousand vaccinations) and in three subjects having received Dryvax® (10.38 events per thousand vaccinations for a combined calculated incidence of 5.97 cases of myo-/pericarditis per thousand vaccinations - 95% confidence interval of 2.87 to 10.95 cases per thousand). These figures represent quite a high rate of potentially life-threatening serious AE following vaccination with a prophylactic vaccine.

Vaccine efficacy data were collected to demonstrate non-inferiority compared to Dryvax® based on the efficacy parameters of major cutaneous reaction ("take") rates and neutralizing antibody titers against VV using a PRNT in both trials. Enrolling vaccinia-naïve subjects in one of the two trials, non-inferiority against Dryvax® could be shown for take rates, but not for antibody titers. On the contrary, for the trial population of vaccinia-experienced subjects enrolled in the second Phase III trial, non-inferiority against Dryvax® could be determined for neutralizing antibody titers, but not for take rates. Taken together, two of the four targeted efficacy measures were met in these trials.

Based on the safety and efficacy data collected in these pivotal Phase III trials, the FDA approved ACAM2000® in September 2007 for use in vaccinia-naïve as well as vaccinia-experienced healthy populations, issuing a black box warning on the prescribing information for the special risks of this conventional second generation smallpox vaccine.

2.4 Origin and Characteristics of IMVAMUNE®

VV is considered the best known member of the poxvirus family and the prototype live viral smallpox vaccine. VV replicates in the cytoplasm of the host cell, its deoxyribonucleic acid does not integrate into the host cell genome and it is non-oncogenic.

Modified Vaccinia Ankara (MVA) was derived from the serial passage of chorioallantois vaccinia Ankara virus (CVA), a VV strain used during the smallpox eradication program. During this passaging, MVA suffered a multitude of mutations within its genome, including six major deletions, resulting in the loss of 15% (31kbp) of original genetic information (Antoine et al. 1998). The deletions affected a number of virulence and host range genes (Antoine et al. 1998; Rosel et al. 1986; Meyer et al. 1991) and as a consequence, MVA exhibits a severely restricted host range in most mammalian cell types (Sutter & Moss 1992; Carroll & Moss 1997; Blanchard et al. 1998; Drexler et al. 1998). Although MVA exhibits a strongly attenuated replication in these cell types, its genes are efficiently transcribed with the block in viral replication being at the level of virus assembly and egress (Sutter & Moss 1992; Carroll & Moss 1997).

IMVAMUNE® has been derived from MVA-572 and is a highly attenuated, purified live vaccine produced under serum-free conditions in chicken embryo fibroblast cells. In contrast to the first and second generation smallpox vaccines IMVAMUNE® is not administered by scarification. The standard route and schedule of IMVAMUNE® are two subcutaneous injections administered four weeks apart. Since IMVAMUNE® is non-replicating in human cells it does not form vesicles (“takes”).

For further details on IMVAMUNE®, please refer to the relevant sections in the Investigator’s Brochure.

2.5 Summary of Preclinical Data with IMVAMUNE®

An extensive nonclinical development program has demonstrated the safety, efficacy and bio-equivalence of IMVAMUNE® compared to other traditional smallpox vaccines. The studies conducted demonstrated the superior attenuation profile of IMVAMUNE® compared to traditional smallpox vaccines (e.g. ACAM2000®, Dryvax®) as well as to other MVA strains. In contrast to other strains, IMVAMUNE® does not replicate in any of the human cell lines tested (Chaplin, Howley, & Meisinger 2002) and is not lethal for severely immune compromised animals (Suter et al. 2009). Repeated administrations (s.c. or intra-muscular (i.m.)) of IMVAMUNE® at doses up to 4.9×10^8 $TCID_{50}$ resulted in injection site irritations and some lymphoid changes; however, these effects were minimal and reversible and are therefore not considered to be dose-limiting.

Two developmental toxicity studies in rats and rabbits demonstrated that none of the tested doses of IMVAMUNE® (1×10^7 $TCID_{50}$ or with 1×10^8 $TCID_{50}$) were teratogenic or caused intrauterine toxicity to the fetuses. In a peri- and postnatal study in rats IMVAMUNE® did not have any effect on the dams or the intrauterine development of the embryos. Furthermore, it did not have any effect on the lactating females or their developing offspring.

Non-clinical studies on immunogenicity and efficacy demonstrated that IMVAMUNE® induces a comparable immune response (antibody and T cells) as traditional smallpox vaccines (ACAM2000®, Dryvax® and Elstree) in both mice and non-human primates (NHP) (Stittelaar et al, 2005).

For more detailed information on preclinical data please refer to the respective sections of the Investigator's Brochure.

2.6 Clinical Profile of IMVAMUNE®

To date, 16 clinical trials (11 sponsored by Bavarian Nordic (BN) thereof 7 under Investigational New Drug (IND) 11596; 5 sponsored by the Division of Microbiology and Infectious Diseases (DMID), National Institute of Allergy and Infectious Diseases (NIAID), National Institutes of Health (NIH) under IND 11229) evaluating the safety and immunogenicity of IMVAMUNE® have been completed or are ongoing. As of April 1, 2012, a total of 3,452 subjects have been vaccinated with IMVAMUNE®, including risk groups with contraindications to conventional smallpox vaccines, such as HIV infected patients and patients with AD.

2.6.1 Safety Overview of IMVAMUNE®

In all completed and ongoing clinical trials, vaccinations with IMVAMUNE® have shown to be generally safe and well tolerated. No cases of death, assessed as being even possibly related, have been reported for a subject in a clinical trial using IMVAMUNE®.

Serious Suspected Adverse Drug Reactions

A total of five (5 out of 3452 vaccinated subjects = 0.145%) serious suspected Adverse Drug Reactions (ADRs) have been reported for IMVAMUNE® so far (see Table 1). All of them have been thoroughly reviewed by BN and the trial specific Data Safety Monitoring Board (DSMB), who concluded that the continued use of IMVAMUNE® in a clinical setting presented no special risks to the subjects. BN assessed reactions as "possibly related" for which no medical cause or medical etiology is known to date and thus a relationship to the vaccine cannot be ruled out technically. No pattern regarding serious suspected ADRs could be detected.

Adverse Drug Reactions

Suspected ADRs, i.e. AEs for which there is a reasonable possible relationship to the trial vaccine, were reported in completed clinical trials POX-MVA-001, -002, -004, -005, -007, -008, -009, -010, -011, -023, -024, -028, HIV-NEF-004 and HIV-POL-002 (n = 2901) as following:

Very common (≥ 10%): vaccination site pain, vaccination site erythema, vaccination site swelling, vaccination site induration, vaccination site pruritus, fatigue, myalgia, headache and nausea.

Common (≥ 1% - < 10%): vaccination site warmth, vaccination site hematoma, vaccination site nodule, rigors/chills, increased body temperature, increased troponin I, pyrexia, appetite disorder, arthralgia, pain in extremity and dizziness.

<u>Uncommon (≥ 0.1% - < 1%):</u> application site anaesthesia, vaccination site movement impairment, vaccination site discoloration, vaccination site haemorrhage, vaccination site inflammation, vaccination site irritation, paraesthesia, rash, vaccination site reaction, lymphadenopathy, tachycardia, vertigo, conjunctivitis, abdominal pain, diarrhoea, dry mouth, vomiting, asthenia, axillary pain, flushing, malaise, oedema peripheral, influenza, nasopharyngitis, sinusitis, upper respiratory tract infection, contusion, hepatic enzyme increased, white blood cell count decreased, back pain, muscle spasm, muscular weakness, musculoskeletal pain, musculoskeletal stiffness, neck pain, paraesthesia, peripheral sensory neuropathy, sleep disorder, cough, pharyngolaryngeal pain, rhinitis, dermatitis, ecchymosis, hyperhidrosis, night sweats, pruritus, rash, urticaria.

The majority of observed suspected ADRs represented local injection site reactions as well as common systemic reactions, typical for modern injectable vaccines and classified as being mild to moderate. More details on frequencies of suspected ADRs according to System of Organ Class and Preferred Term reported so far in completed IMVAMUNE® clinical trials is provided in the current Investigator's Brochure.

Cardiac Signs and Symptoms

Based on observations with first and second generation smallpox vaccines particular attention has been placed on monitoring for cardiac signs and symptoms in all recent clinical trials using IMVAMUNE®.

Despite close cardiac monitoring, no event indicating a case of myo-/pericarditis has been observed in any IMVAMUNE® trial.

Table 1: Serious Suspected ADRs (SAEs Assessed by the Investigator to Be At Least Possibly Related to IMVAMUNE®)

Trial	Subject Age/Gender	Days After Vaccination	Event	Outcome	Underlying Diseases/ Circumstances	Investigator Assessment	BN Opinion
POX-MVA-005	30/Male	70 days after 2nd vaccination	Sarcoidosis	Stable and asymptomatic	Urinary tract infection with Chlamydia trachomatis at time of first symptoms (arthralgia)	Possibly related	Possibly related
POX-MVA-005	31/Female	26 months after 2nd vaccination	Crohn's disease	Stable and asymptomatic under therapy	Abnormal lab results (elevated alkaline phosphatase, absolute neutrophils and platelet counts) at screening for 2-year follow-up study POX-MVA-023 (excluded)	Possibly related	Possibly related
POX-MVA-008	28/Female	8 days after 2nd vaccination	Transitory ocular muscle paresis	Resolved without sequelae	No relevant medical history	Probably related	Possibly related
POX-MVA-010	30/Female	133 days after 2nd vaccination	Congestive heart failure due to cardiomyopathy	Stable under cardiac medications	Surgery for ventricular septal defect as child. HIV infection. Concomitant participation in a Growth-Hormone Releasing Hormone (GH-RH) study (denied, therefore previously unknown to BN); event also assessed as possibly related to GH-RH	Possibly related	Unlikely related
POX-MVA-011	39/Female	1 day after 2nd vaccination	Simple pneumonia and pleurisy	Resolved without sequelae	HIV infection (CD4 count four weeks prior to second vaccination was 299 cells/µl). History of chronic obstructive pulmonary disease. Acute sinusitis and nasal congestion due to swimmer's ear which triggered hospital admittance.	Possibly related	Unlikely related

2.6.2 Immunogenicity Overview of IMVAMUNE®

In three Phase I and II dose finding trials, IMVAMUNE® doses ranging from $1x10^6$ to $1x10^8$ $TCID_{50}$ were tested for safety and immunogenicity (Vollmar et al., 2006, Frey et al., 2007, Von Krempelhuber et al., 2010). Across these trials a linear dose relationship was observed between the vaccine doses and both, ELISA and PRNT titers. Maximum ELISA seroconversion rates and peak titers were reached two weeks after second vaccination, with 100% seroconversion after the second dose for all dose groups receiving at least $2x10^7$ $TCID_{50}$ of IMVAMUNE® or higher. Statistical analysis indicated lower doses ($1x10^6$, $1x10^7$and $2x10^7$ $TCID_{50}$) to be inferior to the $1x10^8$ $TCID_{50}$ dose tested throughout all dose ranging studies, whereas the highest dose tested ($1x10^8$ $TCID_{50}$) achieved ELISA seroconversion rates between 81 and 100% already after the first dose. For the PRNT, the same trend was observed with 71-96% seroconversion rates two weeks after the second IMVAMUNE® administration in all groups receiving the highest dose ($1x10^8$ $TCID_{50}$).

An early onset of seroconversion and the higher titers of total and neutralizing antibodies combined with an excellent safety profile qualified the dose of $1x10^8$ $TCID_{50}$ as the most suitable human dose. Therefore, based on the results of these dose ranging studies, coupled with the animal immunogenicity and efficacy studies, the final optimal (standard) dose and schedule for the general population was decided to be two doses of $1x10^8$ $TCID_{50}$ IMVAMUNE® administered s.c four weeks apart.

Although antibody responses measured by ELISA in HIV infected subjects tend to be lower compared to GMT in healthy and AD subjects two weeks after the first and second vaccinations with IMVAMUNE®, GMTs measured by PRNT were comparable in HIV infected compared to healthy and AD populations. The ability of the second IMVAMUNE® vaccination to significantly boost the immune response in immunocompromised populations to high titer levels is as pronounced as in the healthy population.

In the NIH sponsored trials POX-MVA-002 (DMID 02-017; Frey et al., 2007) and POX-MVA-009 (DMID 06-0012) the immune responses induced by IMVAMUNE® were compared to the traditional smallpox vaccine Dryvax®. In total 97.8% of subjects vaccinated with a single administration of the standard dose of IMVAMUNE® seroconverted by ELISA either at Day 14 or Day 28 post vaccination (29/29 in POX-MVA-002 and 61/63 in POX-MVA-009). 100% of subjects in the Dryvax® group had seroconverted 28 days after scarification (13/13 and 8/8 respectively). A second vaccination with IMVAMUNE® significantly increased the titers measured two weeks later so that the GMTs two weeks after the second vaccination with IMVAMUNE® were comparable to those four weeks after a single vaccination with Dryvax®. In addition, IMVAMUNE® and Dryvax® induced similar levels of T cell immunity with most subjects having detectable T cell responses 26-30 days following vaccinations. Analysis of sera derived from IMVAMUNE® vaccinees compared to sera from Dryvax® recipients demonstrated that subjects vaccinated with IMVAMUNE® had a significantly higher in vitro Variola virus neutralization capacity (titer) compared to subjects vaccinated with Dryvax®. This result supports a comparable efficacy afforded by IMVAMUNE® and traditional smallpox vaccines against smallpox in people (Damon et al., 2009).

Data on cellular immune responses, analyzed in various trials using intracellular cytokine staining for detection of vaccinia-specific Interferon -γ producing CD4+/CD8+ T cells showed a strong, dose-dependency. In vaccinia-experienced subjects, IMVAMUNE® was able

to stimulate the memory T and B cell responses induced by a previous smallpox vaccination with traditional vaccines.

Additional detailed information on the clinical development of IMVAMUNE® is provided in the Investigator's Brochure.

2.7 Rationale

In order to verify the robustness of the GMP production process of the final vaccine product in a clinical setting, consistency trials comparing consecutively produced vaccine lots are deemed appropriate to collect these data as one of the final steps in the development of a vaccine intended to be registered for use in humans.

Respective Guidelines for Industry published by the US FDA and the US Department of Health and Human Services for indications using vaccines for prevention of infectious diseases (e.g. influenza) request lot consistency trials as part of the pivotal Phase III clinical development. Those trials are designed to confirm that there are no significant differences in the biological attributes of various production lots and to bridge potency parameters to clinical immunogenicity. With this pivotal Phase III lot consistency trial BN addresses the need for a lot consistency trial in the development of its third generation smallpox vaccine IMVAMUNE®.

Neutralizing antibodies are considered as the gold standard for predicting protection against smallpox infection and disease and was one of the clinical endpoints accepted by the FDA for approval of the second generation smallpox vaccine ACAM2000® (ACAM 2000 VRBPAC Briefing Document).

Therefore, neutralizing antibody titers at the immune response peak measured two weeks after the second IMVAMUNE® vaccination will be used as primary endpoint in the trial to measure lot consistency of three consecutively produced IMVAMUNE® lots.

In addition, this trial is designed to collect safety data in 3,000 healthy, vaccinia-naïve individuals and therefore to expand the safety data set of IMVAMUNE® obtained from Phase I and II clinical trials. Close monitoring of clinical and laboratory parameters will allow collection of a robust safety data set to confirm and support the so far excellent safety profile of IMVAMUNE®.

This trial will also collect humoral immunogenicity data in vaccinia-naïve subjects using validated PRNT and ELISA to show that the standard scheme with two IMVAMUNE® vaccinations dosed 1×10^8 $TCID_{50}$ can elicit a strong immune response. Since also preclinical data have indicated that there is not a single correlate of protection against lethal challenges with orthopox viruses and the ELISA was generally seen as the better predictor for protection, the ELISA titers will be measured as secondary endpoint.

2.8 Trial Population

Women and men of any ethnicity aged 18 to 40 years who met all the inclusion criteria and none of the exclusion criteria will be recruited for enrollment into this trial.

2.9 Risk/Benefit Assessment

2.9.1 Potential Risks

Blood drawing may cause discomfort, bruising or light-headedness. Rarely, a blood draw may result in infection at the site where the blood is taken.

Preclinical data with IMVAMUNE® in rats and rabbits have revealed no special hazard for humans based on conventional studies of safety.

Based on the present clinical experience with IMVAMUNE® and MVA-based vaccines, adverse reactions to IMVAMUNE® in this trial setting are expected to be comparable to adverse reactions previously reported for IMVAMUNE® and/or those typically seen with other modern vaccines. Main risks involve the development of local reactions at the injection site, e.g. erythema, pain swelling and induration.

As with all injectable vaccines, there is a risk of an allergic reaction or an anaphylactic event, although this has never been observed with IMVAMUNE® or with an MVA-based recombinant vaccine. Trial center staff will watch subjects for at least 30 minutes after each vaccination and in the event that a severe allergic reaction might occur, appropriate medical treatment and supervision will be readily available.

The severe and life-threatening adverse reactions such as progressive vaccinia, eczema vaccinatum, generalized vaccinia and inadvertent inoculation that have been observed after the administration of conventional smallpox vaccines are due to the replication of the vaccinia strains. IMVAMUNE® is replication incompetent in human cells and consequently has a better safety and tolerability profile. It is essentially impossible that IMVAMUNE® could induce the severe side effects listed above associated with replication competent vaccinia viruses. Apart from the better safety profile with regard to severe reactions, the available clinical experience with IMVAMUNE® shows that it is generally better tolerated, for example with regard to local reactions, than conventional smallpox vaccines.

Since this trial enrolls only healthy subjects with no specific underlying disease(s), no subsequent treatment is necessary. Also, no consequent diseases are expected.

2.9.2 Benefits

Trial participants will contribute significantly to the development of a safer smallpox vaccine, which is a benefit to society in view of a potential threat following deliberate release of smallpox virus. Based on the current immunogenicity and efficacy data collected for IMVAMUNE® in preclinical and clinical studies, trial participants (except the trial participants receiving placebo) are expected to acquire protection against smallpox infection.

Future analysis of the samples collected will not directly benefit the subject. BN may learn more about smallpox and other diseases: how to prevent them, how to treat them, or how to cure them.

3 Objectives

Please refer to trial protocol synopsis (see section 1.5).

4 Trial Design

4.1 Experimental Design

This trial is a randomized, double-blind, placebo-controlled Phase III trial to evaluate immunogenicity and safety of three consecutive production lots of IMVAMUNE® (MVA-BN®) smallpox vaccine in healthy, vaccinia-naïve subjects.

In total, four thousand (4,000) vaccinia-naïve subjects will be enrolled in this trial. All subjects will be randomly assigned (1:1:1:1) to one of three IMVAMUNE® groups (Groups 1-3) or the placebo group (Group 4) to receive two vaccinations each with IMVAMUNE® or placebo administered in a double-blind manner.

Group 1 1,000 subjects will receive two s.c. vaccinations with 0.5 ml IMVAMUNE® Lot 1
Group 2 1,000 subjects will receive two s.c. vaccinations with 0.5 ml IMVAMUNE® Lot 2
Group 3 1,000 subjects will receive two s.c. vaccinations with 0.5 ml IMVAMUNE® Lot 3
Group 4 1,000 subjects will receive two s.c. vaccinations with 0.5 ml Placebo (TBS)

4.2 Description of Trial Procedures

The trial procedures will be conducted according to the Trial Procedure Schedule (section 1.6) and as described in this chapter. Visits should be scheduled within the given intervals / visit windows.

4.2.1 Screening Phase

Screening Visit (Day -28 to -1)

All subjects must be thoroughly informed of all aspects of the trial (e.g. trial visit schedule, required evaluations and procedures, risks and benefits) as described in the informed consent form (ICF). The ICF must be reviewed with the subject and signed and dated by the subject and the investigator or person designated by the investigator who conducted the informed consent discussion prior to the initiation of any evaluations or procedures required by the protocol.

After informed consent has been collected, subjects will enter a screening period of up to 28 days before the first vaccination.

**Screening Visit (Days -28 to -1)**
**The following tasks will be performed:**<br>• Subject to read, sign and date ICF<br>• Check vaccination history (previous smallpox vaccination or vaccination with a pox-virus-based vaccine) and absence of smallpox vaccine scar<br>• Check for all inclusion/exclusion criteria<br>• Obtaining medical history and prior/concomitant medications<br>• Complete physical examination including auscultation of heart and lungs and measurement of body weight and height

- Evaluation of vital signs
- Evaluation of family cardiac risk factors
- Calculation of the individual cardiac risk factor (after receipt of lab results for cholesterol)
- Perform baseline ECG
- Counseling on avoidance of pregnancy: Review of acceptable contraceptive methods and recent menstrual history with WOCBP
- Blood draw for safety laboratory (11 ml) including
    - troponin I
    - serum pregnancy test (WOCBP only)
    - cholesterol (total, HDL and LDL)
- Recording and documentation of baseline signs and symptoms

If a subject is screened and cannot be enrolled, because of a certain transient condition (e.g. abnormal lab value due to an acute condition or a missing lab evaluation due to mishandling of the sample), then the subject can be re-screened and the respective test(s) should be repeated as a "partial" re-screening rather than a full re-screening. The re-screening visit must be within the 28 day window started by the first screening visit and the window -28 to -1 before 1st vaccination must not be exceeded. A “partial” re-screening visit is indicated by filling out only the respective re-screening sections of the electronic Case Report Form (eCRF).

If a subject can not be enrolled due to other circumstances (e.g. completion of a wash-out period for a medication or vaccine not allowed during the trial) and the 28 day period is over, a complete re-screening assessment including physical examination, lab examination, and ECG must be performed. The clock then re-starts at the re-screening visit with Day -28 before the first vaccination.

4.2.2 Active Trial Phase

After successfully passing the screening assessments the eligible subject will enter the active trial phase (Visit 1 to Visit 5) starting with Visit 1.

Randomized treatment assignments for the subjects will be done at Visit 1 after re-confirmation of subject’s eligibility. The randomization scheme is 1:1:1:1 (three IMVAMUNE® production lot groups and one placebo group). Randomization will be stratified by CTS. An automated randomization system will be used. The detailed process will be described in a trial specific charter.

The procedures performed at Visit 1 and all following visits are listed below. **Blood draws and all other examinations listed above the vaccination events must always be performed prior to vaccination.**

At Visit 1 / Day 0, subjects will receive the first of two s.c. vaccinations with one standard dose of IMVAMUNE® (0.5 ml vaccine containing a nominal titer of 1 x 10^8 $TCID_{50}$) or placebo containing TBS in the non-dominant upper arm (deltoid region).

Following vaccination, subjects will be kept under close observation by the CTS staff for at least 30 minutes with appropriate medical treatment readily available in case of an unexpected anaphylactic reaction following administration of the vaccine. Any AEs that occur during or after vaccination will be recorded.

Reactogenicity and AEs will be collected on a subject memory aid by having the subject record daily maximum temperatures and solicited AEs for an 8-day period (Days 0-7), beginning with the day of vaccination. The memory aid will be returned to the clinic staff at the following visit. If symptoms persist at Day 7, daily symptoms and temperature will continue to be measured each day until resolved and the last day of symptoms and maximum intensity is recorded on the memory aid. AEs will be assessed at all active trial period visits (Visit 1 to Visit 5).

**Visit 1 (Day 0)**
**Task to be performed prior to randomization and vaccination:**<br>• (Re-) Check of inclusion / exclusion criteria<br>• Targeted physical examination including auscultation of the heart and lungs<br>• Evaluation of vital signs<br>• Recording of concomitant medications<br>• Recording of baseline signs and symptoms and SAEs<br>• Counseling on avoidance of becoming pregnant: Review of acceptable contraceptive methods and recent menstrual history with WOCBP<br>• Urine pregnancy test (WOCBP only)<br>• Blood draw (serum collection, 8 ml) for baseline antibody analysis<br><br>**Blood draw and all tasks mentioned above must always be performed prior to vaccination. If the subject is still eligible for participation in this trial the subject will be randomized. The following tasks will be performed after randomization:**<br>• Administration of first trial vaccination (IMVAMUNE® or placebo s.c.).<br>• Handout of memory aid, ruler and thermometer<br>• Subject observation by CTS staff for at least 30 minutes after vaccination<br>• Recording of immediate AEs/ Adverse Events of Special Interest (AESIs)/SAEs<br>**Temporary deferral of vaccination**: If an acute illness is present, the subject may be vaccinated at a later date within the accepted time window. The vaccine can be administered to persons with a minor illness such as diarrhea, mild upper respiratory infection, or any other mild condition with or without low-grade febrile illness, i.e. oral temperature < 100.4°F (< 38.0°C).

Visit 2 (Visit 1 + 12–16 days)
**The following tasks will be performed:**<br>• Targeted physical examination including auscultation of the heart and lungs<br>• Evaluation of vital signs<br>• Examination of the injection site<br>• Performing ECG<br>• Collection of the memory aid handed out at Visit 1, review with subject<br>• Blood draw for safety laboratory (11 ml) including troponin I<br>• Recording of AEs/SAEs/AESI and concomitant medication

Visit 3 (Visit 1 + 28–35 days)
**Tasks to be performed prior to vaccination:**<br>• Check withdrawal criteria<br>• Targeted physical examination including auscultation of the heart and lungs<br>• Evaluation of vital signs<br>• Counseling on avoidance of becoming pregnant: Review of acceptable contraceptive methods and recent menstrual history with WOCBP<br>• Urine pregnancy test (WOCBP only)<br>• Recording of AEs/SAEs/AESIs and concomitant medications<br><br>**All tasks mentioned above must always be performed prior to vaccination.**<br>**The following tasks will be performed after vaccination:**<br>• Administration of second trial vaccination (IMVAMUNE® or placebo s.c.).<br>• Handout of memory aid<br>• Subject observation by CTS staff for at least 30 minutes after vaccination<br>• Recording of immediate AEs/AESIs/SAEs<br><br>**Temporary deferral of second vaccination**: If an acute illness is present, the subject may be vaccinated at a later date within the accepted time window. The vaccine can be administered to persons with a minor illness such as diarrhea, mild upper respiratory infection, or any other mild condition with or without low-grade febrile illness, i.e. oral temperature < 100.4°F (< 38.0°C).

Visit 4 (Visit 3 + 12–16 days)
**The following tasks will be performed:**<br>• Targeted physical examination including auscultation of the heart and lungs<br>• Evaluation of vital signs<br>• Examination of the injection site<br>• Perform ECG (if clinically indicated)<br>• Blood draw for safety laboratory (11 ml, including troponin I testing if clinically indicated)<br>• Blood draw (serum collection, 8 ml) for antibody analysis<br>• Recording of AEs/SAEs/AESIs and concomitant medications<br>• Collection of the Memory aid handed out at Visit 3, review with subject

Visit 5 (Visit 3 + 28–35 days)
• Targeted physical examination including auscultation of the heart and lungs<br>• Evaluation of vital signs<br>• Urine pregnancy test (WOCBP only)<br>• Recording of AEs/SAEs/AESIs and concomitant medications

4.2.3 Follow-Up (FU) Phase

To monitor long-term safety, the CTS will contact the subject by phone to inquire whether an SAE /AESI might have occurred since the last trial visit and if there is any new information on SAEs/AESIs/AEs ongoing at last trial visit. In cases where a serious condition is detected, the trial subject will be requested to return for a physical examination and further work-up at the CTS.

For subjects who were withdrawn from the 2nd vaccination, the FU visit will be performed 6 months after the 1st vaccination (see section 4.2.5. withdrawal from 2nd vaccination).

Phone FU (Visit 3 + 182–210 days)
• Recording of new SAEs/AESIs and follow-up on ongoing SAEs/AESIs/AEs<br>**If a physical visit at the CTS is deemed necessary, the following should be performed:**<br>• Targeted physical examination including auscultation of the heart and lungs<br>• Evaluation of vital signs<br>• Blood draw for safety laboratory (11ml), if required<br>• Other safety evaluations, if required

4.2.4 Unscheduled Visits

If clinically indicated, additional visits may be necessary between scheduled visits. Unscheduled visits may be scheduled to repeat laboratory testing or physical exams due to a new development. Examinations, performed at unscheduled visits will be documented in the source documents as well as on the respective eCRF pages for unscheduled visits.

4.2.5 Withdrawal from Second Vaccination

The decision not to administer the second vaccination can be made by the investigator or by the subject.

Criteria:

The following criteria should be checked prior to second vaccination.
If any are applicable, the subject should not receive the second vaccination:

- Any clinically significant cardiac sign and symptom (i.e. AESI) defined in Section 8.1.3.3.
- An AE that, in the opinion of the investigator, makes it unsafe for the subject to receive the second vaccination. In this case, the appropriate measures will be taken.
- Anaphylactic reaction following the administration of any vaccine(s).
- Administration of a licensed vaccine not foreseen by the clinical trial protocol.
- Start of chronic administration (defined as more than 14 days) of > 5 mg prednisone (or equivalent) per day or any other immune-modifying drugs.
- Administration of immunoglobulins and/or any blood products.
- Clinical need for concomitant or ancillary therapy not permitted in the trial.
- Use of any investigational or non-registered drug or vaccine other than the trial vaccine.
- Any condition which contradicts administration of the second vaccination in the opinion of the investigator.
- Pregnancy.
- Subject refuses to receive second vaccination.

Procedure:

If the subject did not receive the second trial vaccination the reason for this decision must be recorded in the eCRF and in the subject's medical record. V3 and V4 are not required; and the procedures below should be followed:

- Visit 5 procedures have to be performed within 28 to 35 days after Visit 1.
- FU visit procedures have to be performed 182 to 210 days after Visit 1.

4.2.6 Premature Discontinuation

The trial may be discontinued prematurely for a subject at any time. The decision to discontinue the trial for a subject prematurely can be made by the investigator as well as by the subject himself. Reasons for discontinuing the trial prematurely may include, but are not limited to the following:

Criteria:

- Subject's request to discontinue prematurely (withdrawal of informed consent).
- Subject unwilling or unable to comply with trial requirements.
- Any reason that, in the opinion of the investigator, precludes the subject's further participation in the trial.
- Discontinuation due to an AE.

Procedure:

If a subject discontinues prematurely, the reason for this decision must be recorded in the eCRF and in the subject's medical record. If the subject is unable or not willing to attend all planned visits, every attempt should be made to perform at least a concluding safety visit. For WOCBP a pregnancy test should be performed during this safety visit. If the subject is not willing to undergo any further trial procedure (withdrawal of consent), "withdrawal of consent" needs to be documented as reason for premature discontinuation.

4.2.7 Emergency Unblinding

In case of emergency which makes unblinding necessary (i.e. the subject's safety is dependent on unblinding) a mechanism that permits rapid unblinding but does not permit undetectable breaks of the blinding will allow the investigator the possibility of learning the treatment assignment for a subject. When emergency unblinding is performed via electronic systems such as IVRS and IWRS a backup system enabling unblinding of treatment will be provided.

If the blinding is prematurely broken, it is the responsibility of the investigator to promptly document and explain any unblinding to the sponsor.

The detailed process for emergency unblinding will be described in a trial specific procedure.

4.3 Trial Duration

The total duration of the trial for each subject including the screening period and Phone FU will be up to 39 weeks. The duration of the trial as a whole is dependent on the recruitment period.

4.4 Data Safety Monitoring Board

The DSMB is an independent board that oversees the safety of subjects participating in the trial. The members of the DSMB are independent, i.e. not involved as investigators in any IMVAMUNE® trials and have no direct or indirect financial interests in BN or the contract research organization (CRO) managing the trial. The primary responsibilities of the DSMB are to periodically review and evaluate the accumulated trial data for participant safety, trial conduct and progress, and make recommendations to BN and the Coordinating Investigator and PIs concerning the continuation, modification, or termination of the trial. The DSMB considers trial specific data as well as relevant background knowledge about the disease, test agent, and subject population under trial. DSMB meetings may consist of open sessions (blinded and unblinded participants), closed sessions (unblinded participants only) and executive sessions (DSMB members only). A separate charter describes in detail relevant operational procedures, communication pathways, roles and responsibilities of the DSMB.

If an event occurs which fulfils the trial halting rules the DSMB will review the event in a timely manner and give a recommendation to BN and the Coordinating Investigator and PIs to halt, resume or terminate the trial participation of the affected subject and/or the trial as a whole.

4.5 Trial Halting Rules

A temporary halting or termination for the trial as a whole can be decided in case of an occurrence of

- an SAE
- an unexpected Grade 3 or higher systemic reaction or lab toxicity (see Appendix I)

with an at least reasonable possibility of a causal relationship to the administration of IMVAMUNE®, i.e. the relationship cannot be ruled out.

These parameters are not all-inclusive. Other AEs could occur that would trigger a DSMB review. Any member of the DSMB, the PI and/or the BN Drug Safety (DS) Officer could request a DSMB review based on any observation.

If an event fulfilling the trial halting criteria reaches the investigator's attention, the investigator has the liability to alert the responsible DS Department immediately (within 24 hours) and provide a comprehensive documentation of the event. Contact details of the responsible DS Department are provided in section 8.3.1.

5 Selection of Subjects

Each investigator will keep a log of subjects screened for the trial and provide the reason in case of exclusion. Information about every subject entering the trial will be provided to the CRO managing the trial.

5.1 Recruitment Procedure

Subjects will be recruited actively. Recruitment strategies, including IRB approved paid advertisements, will be evaluated by the sponsor.

Four thousand (4,000) subjects will be enrolled. After signing the ICF, subjects undergo screening procedures to check eligibility according to the inclusion/exclusion criteria. In the event of a screening failure due to mild or limited acute illness or abnormal laboratory values, the subject may be re-screened after resolution of the event. Re-screening may require only an additional blood draw or a complete re-screening evaluation, depending on the circumstances of and the time interval since the initial screening failure. See also section 4.2.1.

5.2 Inclusion Criteria

Please refer to trial protocol synopsis (see section 1.5.)

5.3 Exclusion Criteria

Please refer to trial protocol synopsis (see section 1.5.)

6 Investigational Product

IMVAMUNE® is a highly attenuated live VV (MVA-BN®). It will be provided in liquid-frozen aliquots. One dose of 0.5 ml liquid-frozen vaccine has a nominal virus titer of 1 x 10^8 $TCID_{50}$ MVA-BN®. IMVAMUNE® will be given s.c.

For further details see current version of the Investigator's Brochure.

Placebo consists of the IMVAMUNE® formulation buffer, TBS. It will be provided in liquid aliquots. One dose of 0.5 ml placebo contains 0.605 mg tris (hydroxymethyl)-amino methane and 4.09 mg sodium chloride.

6.1 Production, Packaging and Labeling

IMVAMUNE®:
The bulk drug substance MVA-BN® is produced at Bavarian Nordic A/S and the final drug product IMVAMUNE® is filled and labeled at the contract manufacturer IDT Biologika GmbH.

Addresses:

Bavarian Nordic A/S
Hejreskovvej 10A
3490 Kvistgård, Denmark
Phone: +45 3326 8383

IDT Biologika GmbH
Am Pharmapark
06861 Dessau-Rosslau, Germany
Phone: +49 34901 885 0

Placebo is produced, filled and labeled at Bavarian Nordic GmbH in Berlin, Germany.

Address:

Bavarian Nordic GmbH
Robert-Roessle-Strasse 10
13125 Berlin, Germany

All packages and vials are labeled with the respective label required by the regulatory authorities of the countries in which the trial will be performed.

6.2 Shipment, Storage and Handling

IMVAMUNE® and placebo are packed separately in an open-labeled manner. The vaccine will be shipped temperature controlled from a warehouse to the CTS. The package is handed over to the unblinded personnel in charge of vaccine preparation, e.g. the pharmacist.

At the CTS, only the unblinded personnel have access to the information if a subject is vaccinated with one of the three IMVAMUNE® lots or placebo. They are not allowed to disclose this information.

After receipt of vaccine and placebo, the unblinded personnel are responsible for proper storage.

The liquid-frozen IMVAMUNE® has to be stored at -4°F ± 9°F (-20°C ± 5°C) avoiding direct light. A vial should not be re-frozen once it has been thawed. Details on shipment, storage and handling can be found in BN SOP/CLIN/016 "Storage, Handling and Vaccination Procedures of Liquid Frozen MVA-BN® (IMVAMUNE®) and Recombinant MVA-based Vaccines in Clinical Trials", document number: 10000695.

The placebo (TBS) has to be stored at 36°F to 46°F (+2°C to +8°).

6.3 Preparation, Administration and Dosage

The preparation of the vaccine/placebo will be performed by unblinded personnel only. The unblinded personnel must not be involved in the trial treatment and/or the evaluation of the trial subjects.

Details on vaccine preparation, administration and dosage of IMVAMUNE® is provided in BN SOP/CLIN/016, entitled "Storage, Handling and Vaccination Procedures of Liquid Frozen MVA-BN® (IMVAMUNE®) and recombinant MVA-based vaccines in Clinical Trials", document number: 10000695. Handling of the placebo is in analogy to what is described for IMVAMUNE® after thawing.

6.4 Accountability and Disposal

After receipt of vaccine and placebo, the unblinded personnel have ultimate responsibility for distribution, proper storage and drug accountability of these trial products. Records of receipt, inventory, use by each subject, return or disposal and temperature control must be maintained by the unblinded personnel in the pharmacy file with access restricted to the unblinded personnel.

Used and unused vials should be stored in a safe place and remain the property of BN. The unblinded personnel of the respective CTS are responsible for ensuring adequate accountability of all used and unused investigational medicinal product (IMP). This includes acknowledgement of receipt of each shipment of IMP (quantity and condition) and IMP accountability using an IMP inventory log. The IMP inventory log will document quantity of IMP received, quantity of IMP used for vaccination (including lot number, date dispensed, subject identification number and initials of the person dispensing the IMP) and quantity of IMP returned to the warehouse or destroyed.

Additionally, the quantity of IMP returned to warehouse or destroyed has to be documented on an IMP return/destruction form. In case destruction on site is agreed upon, material should be autoclaved or incinerated and discarded at site according to local regulations.

Furthermore, used syringes should be autoclaved or incinerated and discarded at site according to local regulations.

7 Assessment of Immunogenicity

Immune response analyses are planned at trial Visit 4. The baseline assessment for immunogenicity parameters will be performed on samples taken at trial Visit 1 (before first vaccination). It is planned that antibody serum samples will be taken from all subjects, however only samples from the first 700 subjects per group will be analyzed for immunogenicity endpoints. The remaining samples will be stored for potential future use (see section 7.2).

The methods of collection, storage and handling of lab specimens for immune analysis are specified in the Central Laboratory's Manual, which will be provided to the investigators before enrollment commences. Additionally, training will be provided on the procedures during the Investigator Meeting and/or at the initiation visit.

7.1 Humoral Immunogenicity

Antibody responses against IMVAMUNE® will be measured by means of a validated vaccinia-specific PRNT assay [using Vaccinia Virus Western Reserve (VV-WR) as challenge virus] and a validated vaccinia-specific ELISA (using MVA-BN® as the antigen). The tests will be performed at Bavarian Nordic GmbH, Martinsried, Germany. All clinical analysis personnel will be blinded to subject randomization details.

The protocols for the analytical tests performed are detailed in the SOPs listed in the assay chapters below. The SOPs, effective at the time of trial conduct will be filed in the Trial Master File.

PRNT

The GMT is calculated by taking the antilogarithm of the mean of the log10 titer transformations. Antibody titers below the assay cut-off value will be given an arbitrary value of one (1) for the purpose of calculation.

Seroconversion is defined as:

- Appearance of antibody titers ≥ assay cut-off value in a vaccinia-specific PRNT for initially seronegative subjects.
- An increase of the antibody titer by a factor of at least two-fold compared to the pre-existing baseline titer (at Visit 1) for subjects with a pre-existing antibody titer in the PRNT.

Details on the PRNT procedure can be found in BN SOP/CA/017: "Human Plaque Reduction Neutralization Test Using Vaccinia Virus Western Reserve", document number BN0002807.

ELISA

The GMT is calculated by taking the antilogarithm of the mean of the log10 titer transformations. Antibody titers below the cut-off assay value will be given an arbitrary value of one (1) for the purpose of calculation.

Seroconversion is defined as:

- Appearance of antibody titers ≥ assay cut-off value in a vaccinia-specific ELISA for initially seronegative subjects
- An increase of the antibody titer by a factor of at least two-fold compared to the pre-existing baseline titer (at Visit 1) for subjects with a pre-existing antibody titer in the ELISA.

Details on the ELISA procedure can be found in BN SOP/CA/029: "Automated ELISA for Detection of Vaccinia Specific Antibodies in Human Sera", document number BN0002809.

7.2 Future Use of Lab Specimen

Serum specimens remaining after completion of all immunogenicity testing for the trial will be stored for future analysis supporting the licensure path of IMVAMUNE®. Future analyses will facilitate the bridging of clinical trial immunogenicity data to animal immunogenicity data or to immune response data collected from subjects vaccinated with traditional smallpox vaccines. Subjects will be asked for consenting to storage / future use of samples and will be informed about data protection measures. Specimens will be stored in Bavarian Nordic's secured laboratory area or at an external storage facility in a coded, anonymized manner to ensure data protection. Genetic testing will not be performed.

8 Safety and Reactogenicity

Taking into account the medical history of the subject, safety will be monitored by performing physical examinations including vital signs, routine laboratory measurements and ECGs as well as by evaluating local and general solicited AEs and unsolicited AEs.

Using replication-competent vaccinia-based smallpox vaccines during smallpox vaccination programs in the USA during the last years, cases of acute myocarditis and pericarditis were observed (Cassimatis et al., 2004). Although no such cases have been observed for IMVAMUNE® special cardiac monitoring assessments will be performed.

8.1 Definitions

8.1.1 Medical History

Symptoms present before ICF signature will be documented in the medical history.

8.1.2 Baseline Signs and Symptoms

Any new signs, symptoms or changes in health that occur after ICF signature and before the first vaccination will be recorded in the baseline signs and symptoms sections of the eCRF and in the subject's medical record. Baseline signs and symptoms meeting criteria of an SAE will be reported as outlined in section 8.3.1 "Reporting of SAE".

8.1.3 AE

New signs, symptoms or changes in health starting after the first vaccination are documented in the AE section. AEs are recorded based on unsolicited and solicited questioning.

8.1.3.1 Unsolicited AE

Unsolicited AEs are defined as any untoward (undesirable) occurrence of a medical event in a clinical trial subject temporally associated with the administration of an IMP or a medical

product (MP) which does not necessarily have a causal relationship with this IMP/MP. Up to Visit 5 all AEs (e.g. feeling of ill-health, subjective symptoms and objective signs, intercurrent diseases, accidents, etc.) observed by the investigator and/or reported by the subject must be recorded in the eCRF and in the subject's medical record regardless of the assessment of causality in relationship with the IMP/MP.

Abnormal laboratory values assessed as being clinically significant by the investigator are to be documented as AEs. In addition, abnormal laboratory values fulfilling the Grade 3 or Grade 4 criterion according to the toxicity scale (see Appendix I) are to be documented as AE in the eCRF and in the subject's medical record, regardless of whether they are considered clinically relevant or not. Toxicity grade and seriousness of an AE will be assessed separately, i.e. a Grade 3 or Grade 4 AE will not automatically be regarded as serious.

The investigator should ask the subject if they have experienced any AEs since their last visit. All intercurrent diseases reported by the subject, need to be recorded by the investigator in the appropriate page of the eCRF and in the subject's medical record.

8.1.3.2 Solicited AE

Within this clinical trial protocol solicited AEs are defined as all symptoms specifically listed in the memory aid provided to the subjects following each vaccination. The subjects are requested to monitor and record local symptoms in the memory aid, i.e. erythema, swelling, induration, pruritus and pain at the site of injection as well as general symptoms, i.e. body temperature, headache, chills, myalgia, nausea and fatigue daily for the day of vaccination and the following 7 days (Days 0-7, 8 day duration).

8.1.3.3 AESI

An AESI is defined in this trial as:

- Any cardiac sign or symptom developed since the first vaccination
- ECG changes determined to be clinically significant
- Cardiac enzyme troponin I ≥ 2 x ULN (≥ Grade 2; see toxicity scale, Appendix I)

8.1.3.4 SAE

A SAE is any untoward medical occurrence that at any dose:

- Results in death
- Is life-threatening
  The term "life-threatening" in the definition of "serious" refers to an event in which the patient was at risk of death at the time of the event; it does not refer to an event which hypothetically might have caused death, if it were more severe
- Requires in-patient hospitalization or prolongation of existing hospitalization
- Results in persistent or significant disability or incapacity
- Is a congenital anomaly or birth defect
- or is an otherwise important medical event, e.g.
  - leads to suspicion of transmission of an infectious agent

- suggests lack of efficacy of the product
- documents an overdose of the product

Medical and scientific judgment should be exercised in deciding whether expedited reporting is appropriate in other situations, such as important medical events that may not be immediately life-threatening or result in death or hospitalization but may jeopardize the subject or may require intervention to prevent one of the other outcomes listed in the definition above. These should also usually be considered serious.

Examples of such events are intensive treatment in an emergency room or at home for allergic bronchospasm; blood dyscrasias or convulsions that do not result in hospitalization; or development of drug dependency or drug abuse.

8.2 Assessment

8.2.1 Relevant Medical History

Relevant medical history will be documented at SCR and will focus particularly on any important diseases and in case of infections or tumors, the pathogen involved or the pathological diagnosis, if available. Special attention should be given to history of prior allergic reactions, especially to vaccines.

In addition, smallpox vaccination history must be checked (check for a smallpox vaccine scar and any documentation of previous smallpox vaccination, if available; Note: check also for smallpox vaccination programs during military service or for smallpox response teams or participation in other pox-virus based vaccination trials).

8.2.2 Prior and Concomitant Medications

All concomitant (ongoing) medications except homeopathic substances and dietary supplements must be recorded in the eCRF and in the subject's medical record including information about the indication, dosage regimen, and the onset and end of treatment.

The following medications, taken within 3 months prior to screening, will also be recorded in the eCRF and in the subject's medical record: Vaccines, corticosteroids (via any route of administration), other immune-modulating drugs, immunoglobulins and/or any blood products, investigational drugs and depot preparations which are still active at the date of screening.

8.2.3 Physical Examination and Vital Signs

Complete physical examination:

A complete physical examination will be performed at the SCR. The examination includes a review of major organ systems as well as height and weight. The examination should be directed at finding evidence of any infections, tumors and lymphadenopathy (a grading scale for lymphadenopathy is included in Appendix III). In addition, auscultation of the heart and lungs to check specifically for signs of any heart condition will be performed.

Targeted physical examination:

A targeted physical examination, guided by any signs or symptoms previously identified or any new symptoms that the subject has experienced since the last visit, is required at all visits during the active trial phase (Visit 1 to Visit 5). In addition, auscultation of the heart and lungs will be performed.

A targeted physical examination at the FU Visit is only required if a subject reports during the Phone FU contact any potentially serious condition which will trigger that the trial subject be requested to return for a physical examination and further work-up to the CTS; see section 4.2.3.

8.2.4 Vital signs

Evaluation of vital signs will be performed at screening and at all visits during the active trial phase (Visit 1 to Visit 5). Blood pressure and pulse rate will be taken after the subject has been sitting for two minutes. Body temperature will be measured orally.

8.2.5 Unsolicited AE

All intercurrent diseases reported when the investigator actively inquires the subject will be documented in the source and all required details (e.g. start and stop date, severity) will be assessed. Unsolicited AEs will be reported in the respective section of the eCRF.

AEs will be assessed and documented at all visits of the active trial phase (Visit 1 to Visit 5) and if ongoing at Visit 5 until resolution or until the FU visit at the latest.

SAEs and AESIs will be assessed and documented at all trial visits, including the FU Visit. Ongoing AESIs and SAEs will be followed up until resolution or achievement of stable clinical conditions.

Assessment of Intensity

For all unsolicited AEs not represented in the Toxicity Scale for Laboratory Values (Section 17.1, Appendix I) grading of the maximum intensity will be based on the following descriptions:

Grade 1 An AE which is easily tolerated by the subject, causing minimal discomfort and not interfering with daily activities.

Grade 2 An AE which is sufficiently discomforting to interfere with daily activities.

Grade 3 An AE which prevents daily activities. (Such an AE would, for example, prevent attendance at work and would necessitate the administration of corrective therapy.)

Assessment of Causality

The relationship between the occurrence of an AE and the IMP will be assessed using the categories presented below. For expedited reporting and all other purposes, the categories "none" and "unlikely" will represent no evidence or argument to suggest a causal relationship, while "possible", "probable" and "definite" will be seen to convey that there is evidence or

argument to suggest a causal relationship. Following worst case scenario all AEs without a causality assessment from the investigator will be classified as "possible".

None	• The time interval between the administration of the IMP and the occurrence or worsening of the AE rules out a relationship, **and/or**<br>• another cause is established and there is no evidence of a (concomitant) causal connection with or worsening caused by the IMP.
Unlikely	• The time interval between administration of the IMP and the occurrence or worsening of the AE makes a causal relationship unlikely, **and/or**<br>• the known effects of the IMP or substance class provide no indication of a (concomitant) causal connection with or worsening caused by the IMP and there is another cause which serves as an adequate explanation, **and/or**<br>• although the known effects of the IMP or substance class make it possible to derive a plausible causal chain with regard to a (concomitant) causal connection or worsening, however, another cause is considerably more likely, **and/or**<br>• another cause of the AE has been identified and a (concomitant) causal connection with or worsening caused by the IMP is unlikely.
Possible	• A plausible causal chain with regard to a (concomitant) causal connection with / worsening of the AE can be derived from the pharmacological properties of the IMP or substance class. However, other approximately equally likely causes are known, **or**<br>• although the pharmacological properties of the IMP or substance class provide no indication of a (concomitant) causal connection with / worsening of the AE, there is no other known cause which provides an adequate explanation.
Probable	• The pharmacological properties of the IMP or substance class, **and/or**<br>• the course of the AE after discontinuation of the IMP and possible subsequent re-exposure, **and/or**<br>• specific findings (e.g. positive allergy test or antibodies against the trial drug / metabolites) suggest a (concomitant) causal connection with / worsening of the AE resulting from the IMP, however another cause cannot completely be ruled out.
Definite	• The pharmacological properties of the IMP or substance class **and/or**<br>• the course of the AE after discontinuation of the IMP and possible subsequent re-exposure, **and/or**<br>• specific findings (e.g. positive allergy test or antibodies against the trial drug / metabolites) definitely indicate that there is a (concomitant) causal connection with / worsening of the AE resulting from the IMP and there are no indications of other causes.

8.2.6 Solicited AE

After each vaccination subjects receive a memory aid to record solicited local and general AEs most likely to occur on the day of vaccination and the following 7 days (Days 0-7, 8 day period).

All solicited symptoms observed after vaccination with details concerning the intensity and the course of the reaction should be documented there. The investigator will collect this

information during the following scheduled visits and transfer it to the eCRF. Local and general reactions still ongoing after 7 days will be measured or examined each day until resolution or until no further change can reasonably be expected, and the last day of symptoms and maximum intensity will be documented in the memory aid.

In case of severe and unexpected local and/or general reactions, the subject should be instructed to contact the trial physician outside of scheduled trial visits.

8.2.6.1 Solicited Local AE

The solicited local symptoms erythema, swelling, induration, pruritus and pain at the injection site are to be documented in the memory aid by the subject.

To standardize procedures, uniform rulers will be handed out to all subjects for measurement of erythema, swelling and induration diameters, as will digital thermometers for oral measurements of body temperature.

Assessment of Intensity

Injection site erythema	size measured in diameter
Injection site swelling	size measured in diameter
Injection site induration	size measured in diameter

The maximum severity will be scored as follows:

0	=	0
1	=	< 30 mm
2	=	≥ 30 – <100 mm
3	=	≥ 100 mm

Injection site pruritus:

0	=	Absent
1	=	Mild
2	=	Moderate
3	=	Severe

Injection site pain:

0	=	Absent
1	=	Painful on touch
2	=	Painful when limb is moved
3	=	Spontaneously painful / prevents normal activity

Assessment of Causality

Solicited local AEs are defined as being related to the trial vaccine.

8.2.6.2 Solicited General AE

The solicited general symptoms body temperature, headache, myalgia, nausea, chills and fatigue are to be documented in the memory aid by the subject.

Assessment of Intensity

Subjects are asked to document the solicited general AEs in the memory aid as described in Table 2 below. In the subject's memory aid, the grading of maximum symptom intensity is described in basic, easily understood language based on the following descriptions:

Table 2: Grading of General Symptoms from the Subject's Memory Aid

Medical Dictionary for Regulatory Activities (MedDRA) coded Preferred Term<br>General AEs	Grade	Maximum Severity
Body temperature*	0	< 99.5°F (< 37.5°C)
	1	≥ 99.5 – < 100.4°F (≥ 37.5 – < 38.0°C)
	2	≥ 100.4 – < 102.2°F (≥ 38.0 – < 39.0°C)
	3	≥ 102.2 – < 104°F (≥ 39.0 – < 40.0°C)
	4	≥ 104°F (≥ 40.0°C)
Headache, Myalgia, Nausea, Chills and Fatigue	0	None
	1	Mild: easily tolerated, minimal discomfort and no interference with daily activity
	2	Moderate: Some interference with daily activity
	3	Severe: Prevents daily activity

*Pyrexia is defined as oral temperature ≥ 100.4°F (≥ 38.0 C).

Assessment of Causality

Causal relationship between solicited general AEs and the vaccine will be assessed by the investigator using the same categories as for unsolicited AEs (see section 8.2.5).

8.2.7 Cardiac Assessment

To evaluate the cardiac profile of IMVAMUNE®, targeted physical exams including auscultation of the heart and lung will be performed. Any kind of cardiac signs (i.e. discovered by the physician during examination of the patient) or symptom(s) (i.e. experienced and reported by the patient) detected during the trial such as but not limited to chest pain, dyspnea, arrhythmia or edema are recorded.

ECG

A standard 12-lead ECG will be taken at SCR and at Visit 2. At Visit 4 an ECG is only done

if clinically indicated. ECGs will be evaluated by central ECG reading. The workflow and communication flow will be provided in a separate manual.

Cardiac risk factors
The individual cardiac risk factor is calculated at SCR (for subjects 20 years of age and older), using the National Cholesterol Education Program's risk assessment tool (http://hin.nhlbi.nih.gov/atpiii/calculator.asp?usertype=prof). Subjects with a ten percent or greater risk of developing a myocardial infarction or coronary death within the next 10 years are excluded from trial participation.
In addition, the family cardiac risk factor is evaluated at SCR. Subjects with an immediate family member (father, mother, brother, or sister) who has had onset of ischemic heart diseases before 50 years of age are also excluded from trial participation.

Troponin I
Troponin I will be measured at the SCR and at Visit 2. At Visit 4 troponin I measurement is only done if clinically indicated.

Cardiac events fulfill the definition of an AESI as described under section 8.1.3.3.The investigator will be asked to assess the clinical significance of the case.

Case definitions as published by the Centers of Disease Control and Prevention ("Update: Cardiac-Related Events During the Civilian Smallpox Vaccination Program --- United States, MMWR May 30, 2003, Vol. 52, No. 21, p. 494") are provided in Appendix II in order to:

- help investigators to recognize possible events of acute myocarditis and/or pericarditis and
- distinguish from unspecific and isolated ECG changes without or with unclear clinical significance.

Subjects who develop any kind of cardiac signs or symptoms during the trial such as but not limited to chest pain, dyspnea, arrhythmia or edema are referred to a local cardiologist for cardiac evaluation such as (treadmill) ECG, cardiac enzymes and/or echocardiogram. Depending on the results of these evaluations, further diagnostic tests will be done as recommended by the cardiologist and subjects will be followed up at a frequency determined by the cardiologist. AESIs will be followed up until complete resolution or until the sequelae are stable and considered to be permanent.

Figure 1 outlines the algorithm for assessment of cardiac events.

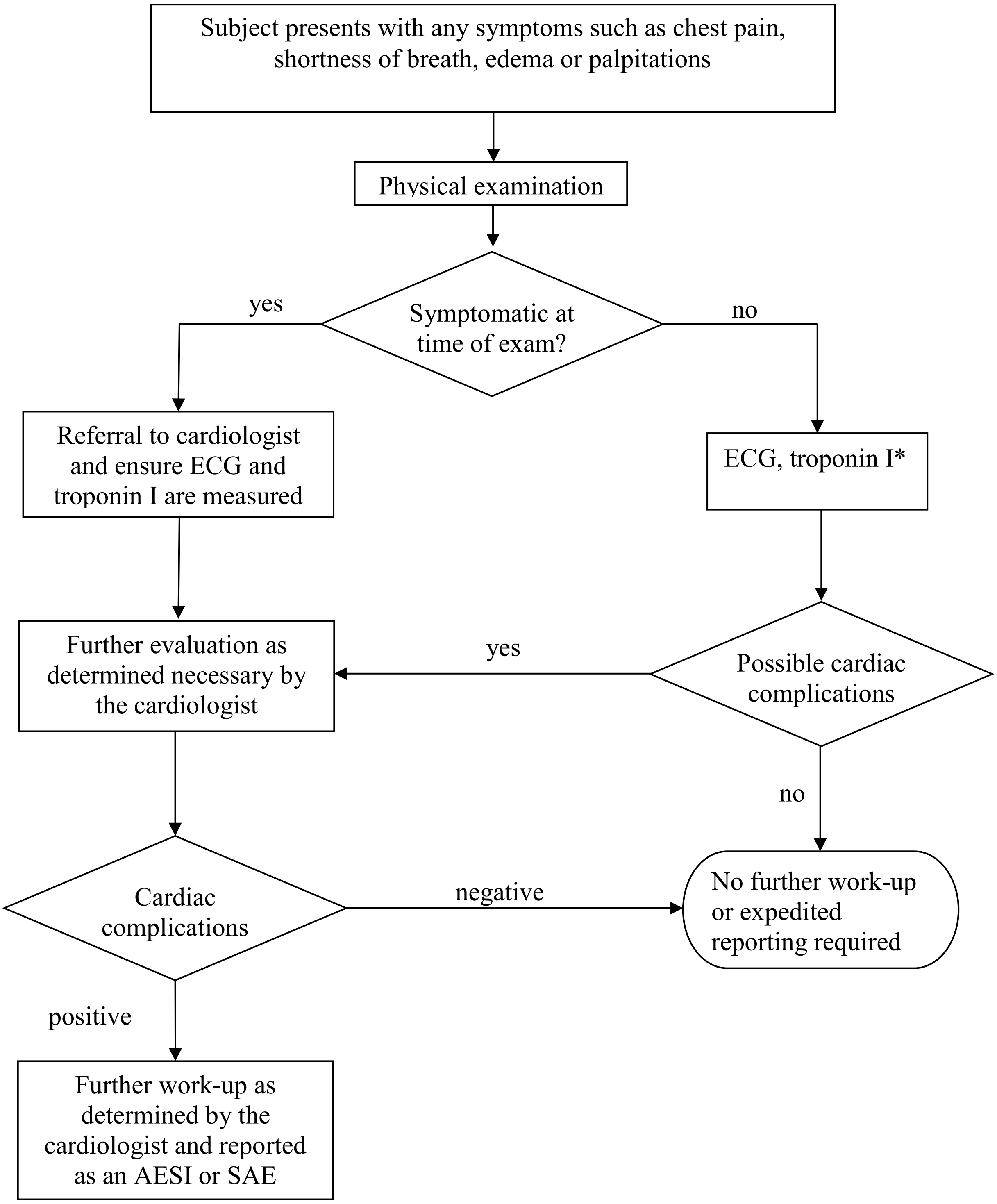

Figure 1: Algorithm for Assessment of Cardiac Events

*At any protocol-scheduled ECG and/or troponin I abnormality, the algorithm will begin at this point.

8.2.8 Safety Laboratory Measurements

The intensity of laboratory / systemic quantitatively measured toxicities will be graded according to the toxicity scale in Appendix I. These grading scales include the laboratory values determined with the routine safety parameters. In case of other laboratory values not included in the routine safety laboratory and not listed in Appendix I, the National Cancer Institute Common Toxicity Criteria table, Version 4.3, published June 14, 2010 will be used for grading of laboratory toxicities.

Safety laboratory is determined at SCR, Visit 2 and Visit 4 and at any other visit(s) if clinically indicated. The safety laboratory measurements are performed at a central laboratory. Laboratory normal ranges are provided by the central laboratory and filed in the Investigator File. Safety laboratory parameters to be evaluated are:

<u>Hematology:</u>
Red blood cell count, hemoglobin, total and differential white blood cell count (WBC), platelet count

<u>Serum chemistry:</u>
Total bilirubin, AP, AST, ALT, serum creatinine (for calculation of CrCl at SCR), sodium, potassium, calcium, troponin I (troponin I mandatory at the SCR and Visit 2 and in addition at Visit 4 if clinically indicated).

<u>Pregnancy test:</u>
A β-human choriogonadotropin (HCG) pregnancy test will be conducted for all WOCBP at SCR, within 24 hours prior to each vaccination (Visits 1, 3) and at Visit 5 (respectively the individual last active trial phase visit). At screening, a serum β-HCG pregnancy test will be performed; all other pregnancy tests will be conducted as urine β-HCG tests.

<u>The following parameters will only be evaluated during SCR for assessment of inclusion / exclusion criteria:</u>

Cholesterol: Total, HDL and LDL

8.2.9 Pregnancy

As per inclusion criteria, women of childbearing potential must have a negative serum pregnancy test at screening and a negative urine pregnancy test within 24 hours prior to each vaccination. In addition, they must have used an acceptable method of contraception for 30 days prior to the first vaccination, must agree to use an acceptable method of contraception during the trial, and must avoid becoming pregnant for at least 28 days after the last vaccination. Nevertheless, IMP exposed pregnancies cannot be excluded with certainty. Subjects who become pregnant prior to the first vaccination will be excluded from the trial and are regarded as screening failure. Subjects who become pregnant during the active trial period (up to and including one month [minimum 28 days] after receiving a dose of vaccine) must not receive additional doses of vaccine but may continue other trial procedures at the discretion of the investigator. All IMP exposed pregnancies should be followed up until delivery.

Subjects should be instructed to notify the investigator if it is determined after completion of the trial that they became pregnant either during the trial or within one month (minimum 28 days) after receiving the last vaccine dose.

8.3 Reporting

8.3.1 Reporting of SAE

All SAEs occurring throughout the entire course of the trial have to be reported to the CRO DS Department. The CTS has to send the completed SAE form by e-mail or fax to the CRO DS Department within 24 hours of becoming aware of the AE.

SAE should be faxed to the following number:

Chiltern Drug Safety
fax: +1 888-726-8416
phone: +1 888-723-2445
email: PVTeam@Chiltern.com

The investigator should not delay reporting because of missing information. Nonetheless, the report should be as complete as possible. This initial notification should include, as a minimum, sufficient information to permit identification of the following:

- the reporter (investigator's name and contact information)
- the subject
- involved trial medication
- AE(s)
- Seriousness criterion and/or criterion for AESI
- date of onset

The CRO DS Department alerts BN DS of all SAEs and provides the available information within 24 hours to BN DS. BN is responsible for expedited as well as periodic reporting to the involved regulatory authorities (e.g. FDA, PEI) according to applicable laws and guidelines. Regulatory authorities will be notified as soon as possible but no later than 7 days after first knowledge of fatal or life-threatening unexpected SAE with an at least possible relationship to the IMP (serious adverse drug reaction [SADR]) and no later than 15 days after knowledge of any other unexpected SADR. In addition BN will report the SAEs to the responsible Biomedical Advanced Research and Development Authority (BARDA) representative when applicable and forward them to the DSMB, while the investigator or the CRO is responsible for reporting to the IECs or IRBs.

Figure 2 outlines the reporting process and timelines SAEs.

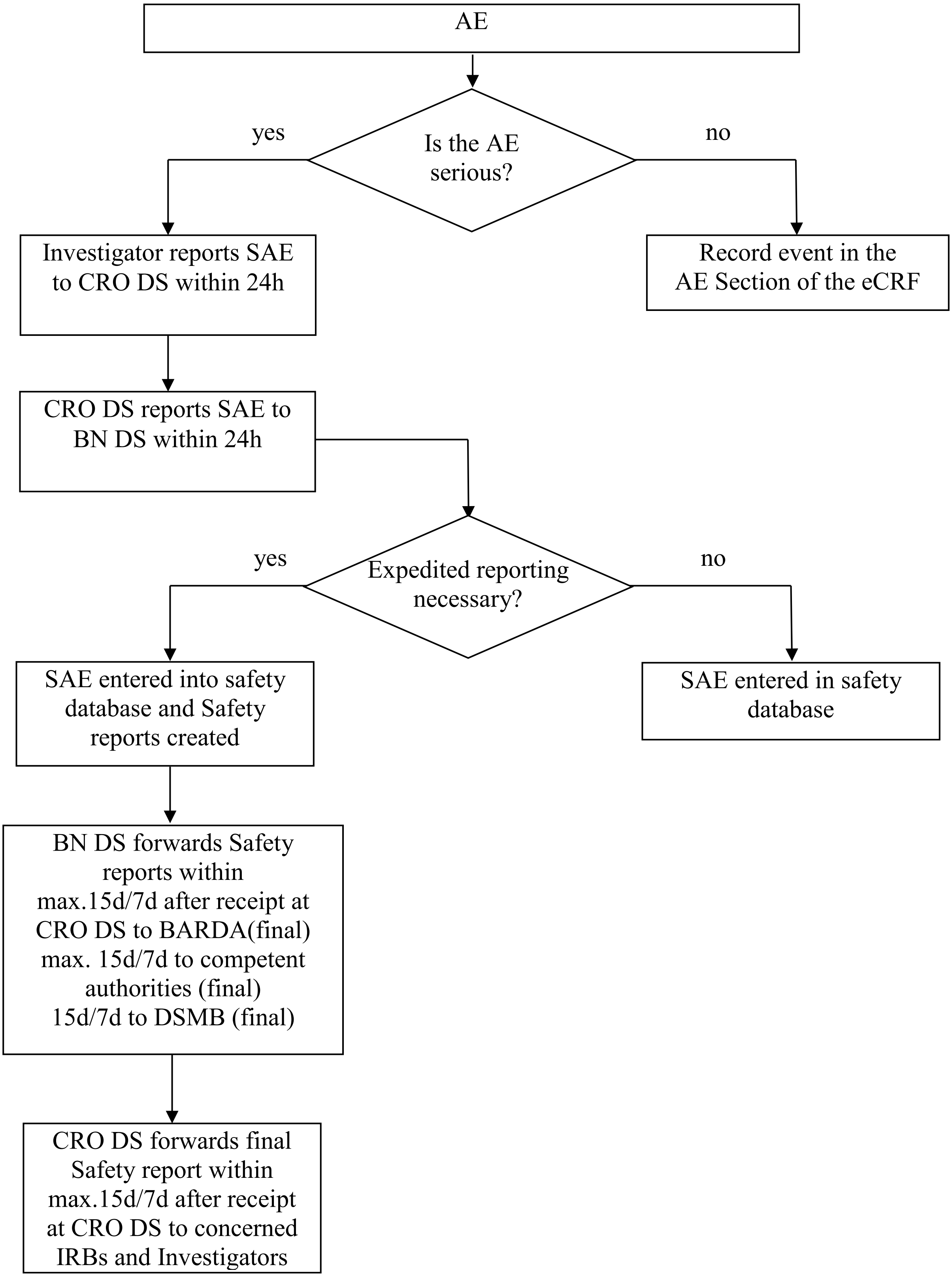

Figure 2: Algorithm for Reporting of SAEs

8.3.2 Reporting of AESI

All AESIs occurring throughout the entire course of the trial have to be reported to the CRO DS Department. The CTS has to send the completed AESI form by e-mail or fax to the CRO DS Department within 24 hours of becoming aware of the AE.

AESIs should be faxed to the following number:

Chiltern Drug Safety
fax: +1 888-726-8416
phone: +1 888-723-2445
email: PVTeam@Chiltern.com

A periodic report for AESIs will be provided from the CRO to BN DS. Figure 3 outlines the reporting process and timelines for AESIs.

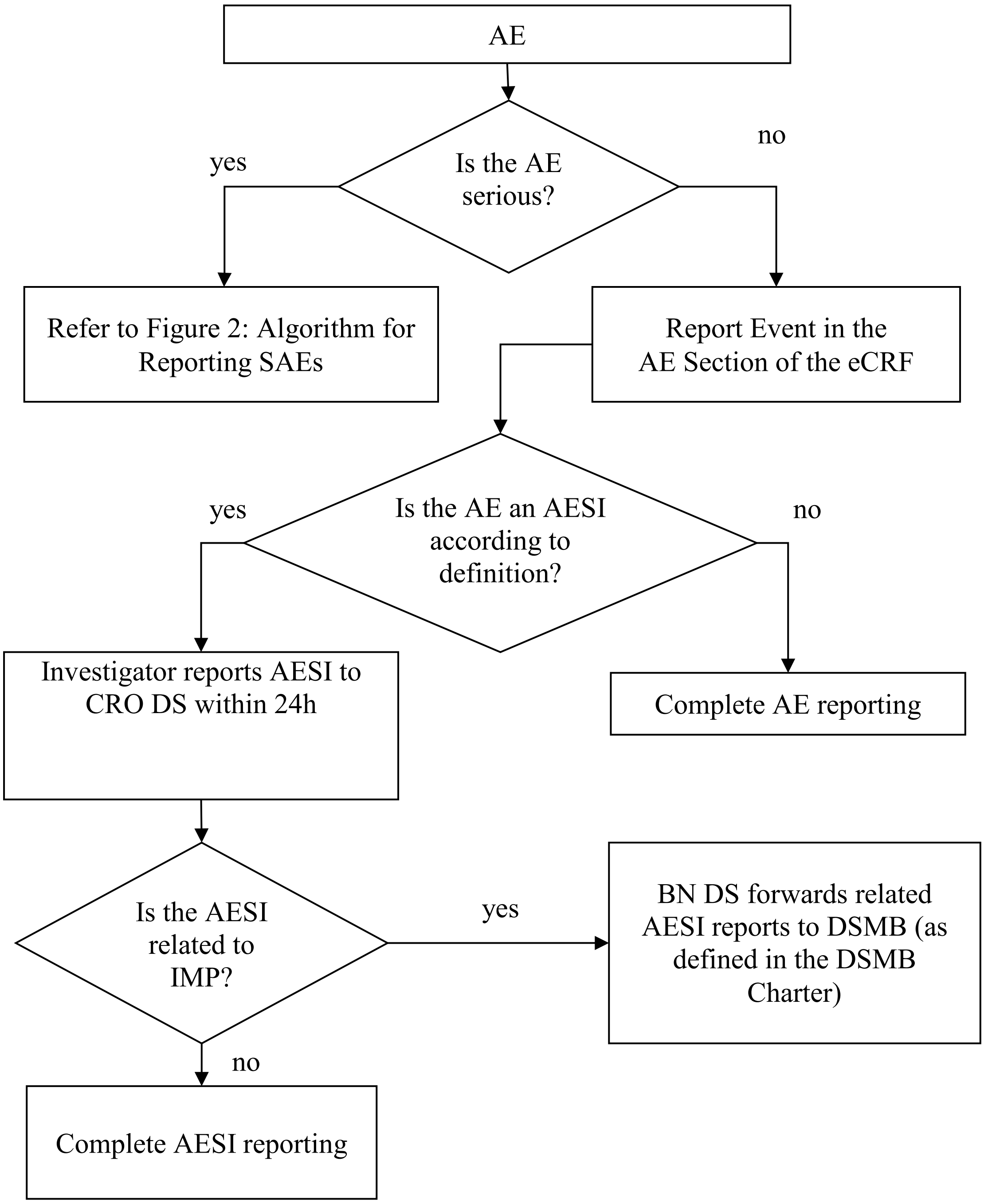

Figure 3: Algorithm for Reporting of AESIs

8.3.3 Reporting of Pregnancies

If a subject becomes pregnant during the active trial period (up to and including one month [minimum 28 days] after receiving a dose of vaccine) this must be reported to BN on a Pregnancy Report Form within 24 hours of the investigator's becoming aware of the event.

A pregnancy should be followed to term, any premature terminations reported, and the health status of the mother and child including date of delivery and the child's gender and weight should be reported to BN after delivery.

Any event during pregnancy fulfilling the criteria for an SAE will be reported as SAE to CRO/BN DS. However, hospitalization for delivery is a prospectively planned hospitalization and is not considered a SAE per se.

9 Statistical Considerations

9.1 Primary Trial Hypothesis

The primary hypothesis of the trial is to show that the humoral immune responses after IMVAMUNE® vaccination are statistically equivalent. The humoral immune response is defined per group as the GMT of a group measured by PRNT at Visit 4. The trial should demonstrate that the means of the log10 titers are equivalent within a pre-specified amount. This amount is called the margin of equivalence (Δ).

Suppose m1 is the mean of the log10 titers in Group 1, m2 is the mean of the log10 titers in Group 2 and m3 is the mean of the log10 titers in Group 3. The test on equivalence will be applied for the following hypothesis:

H0: $m1 - m2 \leq -\Delta$ OR $m1 - m2 \geq \Delta$ OR $m1 - m3 \leq -\Delta$ OR

$m1 - m3 \geq \Delta$ OR $m2 - m3 \leq -\Delta$ OR $m2 - m3 \geq \Delta$

versus

H1: $m1 - m2 > -\Delta$ AND $m1 - m2 < \Delta$ AND $m1 - m3 > -\Delta$ AND

$m1 - m3 < \Delta$ AND $m2 - m3 > -\Delta$ AND $m2 - m3 < \Delta$

Δ is the margin of equivalence and is chosen to be 0.301 for the log 10 titers of the PRNT (this is equivalent to a factor of 2 for the GMT).

From previous experience it is anticipated that the standard deviation (SD) for the log10 titers for PRNT in the IMVAMUNE® groups is 0.85.

The above hypothesis will be tested based on the difference of the log10 titer means being approximately normally distributed (which has been shown to be a very good approximation in all previous IMVAMUNE® studies). Specifically, the lower and upper one-sided 97.5% confidence interval limits for the difference of log10 means will be calculated. If all of the lower confidence interval limits for m1–m2, m1–m3 or m2–m3 are above –Δ and all of the lower confidence interval limits for m1–m2, m1–m3 or m2–m3 are below Δ the null-hypothesis will be rejected in favor of the alternative hypothesis of equivalence.

9.2 Endpoints

Please refer to trial protocol synopsis (see section 1.5).

9.3 Sample Size Calculation

Primary objective

The primary objective of the trial is to demonstrate equivalence of three consecutively produced lots of IMVAMUNE® in terms of humoral immunogenicity in vaccinia-naïve subjects after two doses. Assuming a significance level of 5%, an analyzable sample size of 600 in each group for the per protocol set, and an expected log10 titer SD of 0.85 in all groups for the PRNT, then the primary hypothesis of the GMT based on the PRNT at Visit 4 (i.e. after two IMVAMUNE® vaccinations) has a power of >80% of showing equivalence for all three IMVAMUNE® groups, using an equivalence margin of Δ=0.301 on the log10 scale.

In order to account for a dropout rate of about 15%, which has been observed in previous IMVAMUNE® trials, blood will be analyzed from a total of 700 subjects in each group.

In order to allow a proper safety analysis as described in the sections below, 300 additional subjects will be enrolled in each group to ensure that a total of 1,000 subjects per group are available.

Secondary objectives

A safety sample size of 1,000 in each of the three IMVAMUNE® groups will give a combined IMVAMUNE® safety population of 3,000 subjects. With this sample size the trial will have a 95% chance of detecting any uncommon AEs, i.e. with an incidence of at least 1/1,000.

An important cardiac safety objective of this trial is to demonstrate that the incidence of myo-/pericarditis cases in the combined IMVAMUNE® trial groups (Groups 1–3) is less than 1/330 (lower 95% confidence limit of the 10 cases out of 1,675 subjects observed in the ACAM2000® development program). If no cases of myo-/pericarditis are observed in the 3,000 IMVAMUNE® subjects then the true incidence of myo-/pericarditis will be significantly (alpha=0.05) below the 1/1,000 rate. In contrast, if one or two cases of myo-/pericarditis are observed in the three combined IMVAMUNE® trial groups then the observed rate is significantly below 1/330 (alpha=0.05), which is the most optimistic rate that is likely for the incidence of ACAM2000®.

It is also relevant to compare the observed incidence of myo-/percarditis cases in the combined IMVAMUNE® trial groups to that seen in the placebo group (Group 4). Hence cardiac monitoring will be performed in all 1,000 subjects of every trial group (including the placebo group) two weeks after each IMVAMUNE® / placebo administration.

The background incidence of myo-/pericarditis from literature is assumed to be 1–5 cases per 100,000 in the healthy population (Karjalainen and Heikkilä, 1999), which represents the incidence expected following vaccination with IMVAMUNE®. If we assume that the background rate of the healthy population is at the upper range of this estimate, i.e. 0.00005 (5/100,000), then the chance of observing a single case of myo-/pericarditis in the combined IMVAMUNE® groups is 13.9%. Therefore, the observation of a single case of myo-/pericarditis would not be a significant event. The observation of 2 or more cases of myo-

/pericarditis would occur with a probability of only 1.02%, and hence would be an unlikely event.

In summary, if no case of myo-/pericarditis would be observed in the placebo group and no cases in the 3,000 subjects of the combined IMVAMUNE® trial groups then it can be concluded that the incidence is statistically significantly lower than 1/1,000. Alternatively, if a single case would be observed in the combined IMVAMUNE® trial groups then the rate is still statistically significantly lower than 1/330 (which is the best case scenario for ACAM2000®), and is not significantly different from the assumed background incidence rate of 5/100,000. Finally, if two cases would be observed then this is still significantly lower than 1/330, but is significantly higher than the background incidence of 5/100,000.

However, the chance of observing a single case of myo-/pericarditis in the 1,000 placebo subjects is only 4.9% (assuming the background rate is 0.00005, Karjalainen and Heikkilä, 1999). Hence the observation of a single case of myo-/pericarditis in the placebo group would be significant evidence that the baseline rate in the healthy trial population is actually higher than 0.00005.

Careful cardiac monitoring of all subjects will also allow comparison between groups with regard to the incidence of other cardiac events such as ECG changes, which are expected to be observed in all trial groups, including placebo subjects. A completed Phase II IMVAMUNE® trial enrolling 745 subjects included close cardiac monitoring and revealed several cases of changes in cardiac status in a young, healthy population. Most of these cardiac observations were assessed to be clinically not significant and similarly distributed amongst all trial groups including the placebo group. These data demonstrate the necessity to include a placebo arm for filtering out background pathology in healthy subjects not receiving IMVAMUNE®.

9.4 Trial Cohorts/Datasets to be Evaluated

For the statistical analysis the included subjects will be divided up into the following datasets:

<u>Full Analysis Set (FAS):</u>

This is the subset of subjects who received at least one dose of trial vaccine and for whom post-vaccination safety data are available.

The main analysis of safety will be performed on this analysis set.

<u>Per Protocol Set (PPS):</u>

This is a subset of subjects in the FAS who additionally adhere to all protocol conditions. Minor protocol violators can, however, be included into this dataset.

The decision whether a protocol deviation is major or not for the classification of subjects to subsets will be made case-by-case in a blinded data review meeting before data base closure.

The primary endpoint dataset will be the PPS. All confirmatory testing is based on this subgroup. For further descriptive purposes, the same statistical procedures will be applied to the FAS.

9.5 Biometrical Evaluation

As soon as the last subject has completed the FU visit and after any necessary settlement of queries etc. in the eCRFs, data will be locked. A full analysis of the data available will be performed.

All data obtained in this trial and documented in the eCRFs will be listed. For parameters of interest, summary tables with descriptive group statistics for metrical variables will be prepared. For categorical / dichotomous variables summary tables showing the absolute and relative count in each category will be prepared. Summaries will be presented for Groups 1, 2, 3 and 4 separately and for Groups 1, 2 and 3 combined.

Full details of the analyses will be defined in a Statistical Analysis Plan which will be finalized prior to database lock.

Chiltern will be responsible for data management and statistical evaluation. Data will be analyzed using SAS® software. The procedure for accounting for missing, unused and spurious data will be given in the Statistical Analysis Plan.

All statistical tests for secondary endpoints and comparisons are regarded as descriptive and no adjustment for multiple testing will therefore be done.

The occurrence of solicited local and general AEs at the day of vaccination and the following 7 days (Days 0–7 = 8 day period) will be summarized on a per subject and per vaccination basis.

Unsolicited AEs will be coded using MedDRA coding terminology. The intensity of AEs will be graded according to section 8.2.5.

SAEs will be listed separately. Each SAE will be described individually in detail.

Clinical laboratory test results will be marked whether the result is below, within or above the respective reference range. The number of values outside of the corresponding reference range will be counted.

All ECGs will be evaluated according to a centralized procedure as described in the ECG Assessment Plan. Detailed descriptive analyses of the reasons (category) of abnormalities will be provided.

10 Ethical Aspects

10.1 Ethical and Legal Regulations

The PIs are to ensure that this clinical trial is conducted in complete accordance with the provisions of the 1996 version of the Declaration of Helsinki, the national laws and other guidelines for the conduct of clinical trials like ICH GCP to guarantee the greatest possible subject protection.

10.2 Approval by an IEC / IRB

The protocol must be reviewed by the competent IEC / IRB according to the national laws of the respective CTS before the first subject is screened for this trial.

If one of the investigators is a member of one of these committees, he/she may not vote on any aspect of the review of this protocol.

The Sponsor will assure that the IEC / IRB is informed of any amendment to the protocol and any unanticipated problems involving risks to human subjects included in the trial. Such information will be provided to the IEC / IRB at intervals appropriate to the degree of subject risk involved, but not less than once a year. Copies of all correspondence between the investigator and the IEC / IRB must be forwarded immediately to the Sponsor. In case of withdrawal of IEC / IRB approval of the trial, the Sponsor has to be contacted immediately by facsimile, e-mail or telephone.

10.3 Confidentiality and Data Protection

The PI of the respective CTS is obliged to ensure anonymity of the subject. He/she has to make sure that all documents including eCRFs provided (e.g. in the course of a marketing authorization procedure) to third parties (in this case: to the manufacturer of IMVAMUNE® or to an authority) contain no subject names.

Only a subject and center number may identify subjects; their name or clinic and subject's medical record number may not be used. The PI keep separate confidential subject logs for trial enrollment which allows subject numbers to be matched with names and addresses of subjects at any time. Documents not meant to be passed on to third parties have to be stored confidentially by the PI.

Any information collected in the course of the trial may be made available only to persons directly involved in this trial (PI and his staff members, monitors, statisticians) or to authorized persons by the Sponsor or the PI or authorities. The Sponsor of the trial will only receive pseudonymized data for analysis.

11 Informed Consent

No subject can participate in this trial without having given informed consent in writing after the investigator or his delegate has informed the subject clearly and completely, verbally and in writing, over the purpose, procedures, the potential future use of blood samples and potential benefits and risks of the trial prior to any trial specific procedure.

One signed copy of the informed consent including HIPAA must be given to each subject and one signed copy must remain in the investigator site file and be available for verification by the monitor, sponsor auditor or competent regulatory authorities at any time.

Subjects must be informed unequivocally that they may refuse participation in the trial and that they may withdraw from the trial at any time and for whatever reason and that withdrawal of consent will not affect their subsequent medical treatment or relationship with the treating physician.

Subjects also consent to authorize the monitor, quality assurance personnel and regulatory authorities to inspect source documents for data verification and quality assurance purposes. Such verifications will always be conducted at the CTS and under the ethical supervision of the investigator. All aspects of the confidentiality of the subject's data will be guaranteed.

The informed consent form will be prepared in accordance with ICH GCP guidelines and must be approved by the appropriate IEC / IRB.

12 eCRFs and Retention of Records

12.1 eCRF

In this trial, the use of an eCRF is planned.

All eCRFs are to be filled out completely by the trial personnel, then reviewed and signed electronically by the PI to confirm their correctness in a timely manner.

It is the PIs responsibility to ensure that all subject data entered including discontinuations or changes in trial or other medications in the eCRF are accurate and supported by the subject's medical records unless the eCRF has been declared as source documentation by BN. The eCRFs for any subject leaving the trial should be completed at the time of the final visit or shortly thereafter.

12.2 Retention of Records

Essential documents as listed in ICH GCP need to be archived according to ICH GCP and national law, whatever is longer.

To meet regulatory requirements, the original source data and an electronic copy of the eCRF data will be stored at the CTS. All eCRF data will be stored and archived according to the Clinical Data Interchange Standards Consortium (CDISC) Operational Data Modeling (ODM) (see www.cdisc.org for details). Since CDISC ODM is also the source for the Electronic Data Capture web-based system, no transcription of data is necessary. If needed, paper copies (file printouts) can be created from the ODM file.

13 Monitoring of the Trial

The CRO (contact information to be found in the "Responsibilities" section in the beginning of this protocol) will be contracted to perform monitoring services according to ICH-GCP.

Monitoring will be conducted according to the monitoring plan which must be approved by BN and the CRO. The monitoring plan will specify in detail the items for source data verification and other tasks to be performed by the clinical research associate (CRA) during the visit at the CTS.

The CRA is responsible for obtaining an overview of the course of the trial in co-operation with the investigators, checking if the clinical trial protocol is being observed, and helping the investigators to solve any problems which may arise. All documents in the context with this clinical trial will be handled confidentially at all times.

The PI has agreed to give the CRA access to relevant hospital or clinical records to confirm their consistency with the eCRF entries and to obtain an adequate overview of the course of the trial. The CRA verifies that the entries in the eCRF are complete, accurate and supported by source documents. In addition the CRA will verify that all required data documented in the source were transferred accurately in the eCRF. This will be done under preservation of data protection.

The source data verification must be performed by direct insight in the subject's medical record. If a subject refuses to consent to this procedure, he/she may not be enrolled in the trial. CTS will provide direct access to all trial related data for the purpose of monitoring and auditing by local and regulatory authorities. The PI (or a representative) has further agreed to support the monitor in solving any problems he/she discovers during his/her visits.

14 Audits and Inspections

Audits and inspections may be carried out by the quality assurance department (BN and/or CRO), local authorities, or authorities to whom information on this trial has been submitted. All documents pertinent to the trial must be made available for such audits / inspections. Informed consent of subjects participating in this trial has to include the consent in this access to source documents.

15 Responsibilities of the Investigator

The PI agrees to carry out the trial in accordance with the guidelines and procedures outlined in this clinical trial protocol. The PI especially consents to strictly adhere to the ethical principles (see section 10 of this protocol).

Changes to the protocol require written "Amendments to the protocol" and written approval by the Coordinating and the PI of the respective CTS. Changes are allowed only if trial value is not reduced and if they are ethically justifiable. The amendment must be passed on to all participating PIs with the obligation to adhere to its provisions. If warranted, the subject information has to be changed accordingly.

It is within the responsibility of the PI that the eCRF has to be completed in a timely manner after each subject visit and electronically signed after the subject has finished the trial for each subject participating in the trial.

At the conclusion of the trial, the investigator will return all partly used, unused and empty vaccine vials to the Sponsor or the vaccine vials will be destroyed on the CTS.

The investigator may ask to terminate the trial due to administrative or other reasons. If this should be the case, appropriate measures which safeguard the interests of the participating subjects must be taken after verification and consultation with the PI.

Each investigator will maintain appropriate medical and research records for this trial, in compliance with ICH E6 (R1) Guideline for GCP, Section 4.9, and regulatory and institutional requirements for the protection of confidentiality of subjects. He/she will permit authorized representatives of the sponsor and regulatory agencies to review (and, when required by applicable law, to copy) clinical records for the purposes of quality assurance reviews, audits, and evaluation of the trial safety and progress.

The PI agrees to follow the detailed publication policy included in the clinical trial agreement.

By signing this protocol, the PI confirms that he/she has read the entire clinical trial protocol, agrees to its procedures, and will comply strictly with the formulated guidelines.

16 References

ACAM 2000 Vaccines and Related Biological Products Advisory Committee (VRBPAC) Briefing Document, April 2007 http://www.fda.gov/ohrms/dockets/ac/07/briefing/2007-4292B2-00-index.htm

Antoine G, Scheiflinger F, Dorner F, & Falkner FG (1998). The complete genomic sequence of the modified vaccinia Ankara strain: comparison with other orthopoxviruses.Virology 244, 365-396.

Blanchard TJ, Alcami A, Andrea P, & Smith GL (1998) Modified vaccinia virus Ankara undergoes limited replication in human cells and lacks several immunomodulatory proteins: implications for use as a human vaccine.J.Gen.Virol. 79 (Pt 5), 1159-1167.

Cassimatis DC, Atwood JE, Engler RM, et al. 2004. Smallpox vaccination and myopericarditis: a clinical review. J. Am. College. Cardiol.; 43:1503-1510.

Carroll MW & Moss B (1997) Host range and cytopathogenicity of the highly attenuated MVA strain of vaccinia virus: propagation and generation of recombinant viruses in a nonhuman mammalian cell line.Virology 238, 198-211.

Chaplin PJ, Howley P, Meisinger C. Modified Vaccinia Ankara Virus Variant. Copenhagen patent WO 02/42480 A2. 2002 May 2002.

Damon IK, Davidson WB, Hughes CM, et al. Evaluation of smallpox vaccines using variola neutralization. J.Gen.Virol. 2009 Aug;90 (Pt 8):1962-6.

Drexler I, Heller K, Wahren B, Erfle V, & Sutter G (1998) Highly attenuated modified vaccinia virus Ankara replicates in baby hamster kidney cells, a potential host for virus propagation, but not in various human transformed and primary cells.J.Gen.Virol. 79 (Pt 2), 347-352.

Frey, SE, Couch, RB, Tacket, CO, Treanor, JJ, Wolff, M, Newman, FK, Atmar, RL, Edelman, R, Nolan, CM, and Belshe, RB (2002) Clinical responses to undiluted and diluted smallpox vaccine. N.Engl.J.Med., 346[17], 1265-1274. 2002.

Frey SE, Newman FK, Kennedy JS, et al. Clinical and immunologic responses to multiple doses of IMVAMUNE® (Modified Vaccinia Ankara) followed by Dryvax® challenge. Vaccine 2007; 25 (51):8562-73.

Goldstein JA, Neff JM, Lane JM, & Koplan JP (1975) Smallpox vaccination reactions, prophylaxis, and therapy of complications.Pediatrics 55, 342-347.

Guillaume JC, Saiag P, Wechsler J, Lescs MC, & Roujeau JC (1991) Vaccinia from recombinant virus expressing HIV genes. Lancet 337, 1034-1035.

Karjalainen J and Heikkilä J (1999). Incidence of three presentations of acute myocarditis in young men in military service. European Heart Journal 20, 1120-1125

Lane JM, Ruben FL, Neff JM, & Millar JD (1969) Complications of smallpox vaccination, 1968.N.Engl.J.Med. 281, 1201-1208.

Lane JM, Ruben FL, Neff JM, & Millar JD (1970) Complications of smallpox vaccination, 1968: Results of ten statewide surveys.J.Infect.Dis. 122, 303-309.

McCurdy LH, Larkin BD, Martin JE, & Graham B (2004) Modified Vaccinia Ankara: Potential as an Alternative Smallpox Vaccine, CID 1749-1753

McElwain WP (1972) Complications of smallpox vaccination.J.Ky.Med.Assoc. 70, 165-166.

Meyer H, Sutter G, & Mayr A (1991) Mapping of deletions in the genome of the highly attenuated vaccinia virus MVA and their influence on virulence.J.Gen.Virol. 72 (Pt 5), 1031-1038.

Redfield RR, Wright DC, James WD et al. (1987) Disseminated vaccinia in a military recruit with human immunodeficiency virus (HIV) disease.N.Engl.J.Med 316, 673-676.

Rosel JL, Earl PL, Weir JP, & Moss B (1986) Conserved TAAATG sequence at the transcriptional and translational initiation sites of vaccinia virus late genes deduced by structural and functional analysis of the HindIII H genome fragment.J.Virol. 60, 436-449.

Stittelaar KJ, van AG, Kondova I et al. (2005) Modified vaccinia virus Ankara protects macaques against respiratory challenge with monkeypox virus. J Virol 79(12):7845-7851.

Suter M., Meisinger-Henschel C., Tzatzaris M., Hülsemann V., Lukassen S., Wulff N. H., Hausmann J., Howley P., & Chaplin P. (2009) Modified vaccinia Ankara strains with identical coding sequences actually represent complex mixtures of viruses that determine the biological properties of each strain. Vaccine 27, 7442-7450.

Sutter G & Moss B (1992) Nonreplicating vaccinia vector efficiently expresses recombinant genes.Proc.Natl.Acad.Sci.U.S.A 89, 10847-10851.

Vollmar, J et al. (2006) Safety and Immunogenicity of IMVAMUNE: A promising candidate as a third generation smallpox vaccine.Vaccine 24, 2065-2070.

Von Krempelhuber A, Vollmar J, Pokorny R, et al. (2010) A randomized, double-blind, dose-finding Phase II study to evaluate immunogenicity and safety of the third generation smallpox vaccine candidate IMVAMUNE.Vaccine 28(5), 1209-1227.

17 Appendices

17.1 Appendix I: Toxicity Scale for Laboratory Values

Grade 1 or Grade 2 toxicity is only graded according to Table 3 and Table 4, if the value is outside of the institutional normal range applicable for this trial.

Estimating severity grade
For abnormalities NOT found elsewhere in the Toxicity Tables use the scale below to estimate grade of severity:

Grade 1 An AE which is easily tolerated by the subject, causing minimal discomfort and not interfering with daily activities.

Grade 2 An AE which is sufficiently discomforting to interfere with daily activities.

Grade 3 An AE which prevents daily activities. Such an AE would, for example, prevent attendance at work and would necessitate the administration of corrective therapy.

Grade 4 Life-threatening or disabling

Serious or life-threatening AEs
ANY clinical event deemed by the clinician to be serious or life-threatening should be considered a Grade 4 event. Clinical events considered to be serious or life-threatening include, but are not limited to: Seizures, coma, tetany, diabetic ketoacidosis, disseminated intravascular coagulation, diffuse petechiae, paralysis, acute psychosis, severe depression.

Table 3: Toxicity Scale for Serum Chemistry

Lab Value	Mild (Grade 1)	Moderate (Grade 2)	Severe (Grade 3)	Potentially Life Threatening (Grade 4)
Sodium – Hyponatremia mmol/L	< Lower Limit of Normal (LLN) – 132	130 – 131	125 – 129	< 125
Sodium – Hypernatremia mmol/L	> ULN – 149	150 – 154	155 – 159	≥ 160
Potassium – Hyperkalemia mmol/L	> ULN – 5.9	6.0 – 6.5	6.6 – 7.0	> 7.0
Potassium – Hypokalemia mmol/L	< LLN – 3.1	2.5 – 3.0	2.0 – 2.4	< 2.0
Calcium – Hypercalcaemia mmol/L	> ULN – 2.89	2.90 – 3.09	3.10 – 3.30	> 3.30
Calcium- Hypocalcaemia mmol/L	< LLN – 2.00	1.76 – 2.00	1.50 – 1.75	< 1.50
Serum creatinine mg/dl	>ULN – <1.5 x ULN	≥ 1.5 – < 3 x ULN	≥ 3.0 – 6.0 x ULN	> 6.0 x ULN
Alkaline Phosphatase increase by factor	> 1.25 – <2.0 x ULN	≥ 2.0 – < 3.0 x ULN	≥ 3.0 x ULN	
Liver Function Tests increase by factor	> 1.0 – < 2.5 x ULN	≥ 2.5 – < 4 x ULN	≥ 4.0 x ULN	
Total Bilirubin increase by factor	> ULN – 1.5 x ULN	> 1.5 – 3.0 x ULN	> 3.0 – 10.0 x ULN	> 10.0 x ULN
Cardiac troponin I increase by factor	>ULN – <2.0 x ULN	≥ 2.0 – < 5.0 x ULN	≥ 5.0 x ULN	
Total Cholesterol mg/dl	> ULN – 300	> 300 – 400	> 400	

Table 4: Toxicity Scale for Hematology

Lab Value	Mild (Grade 1)	Moderate (Grade 2)	Severe (Grade 3)	Potentially Life Threatening (Grade 4)
Hemoglobin (Female) g/dl	< LLN – ≥ 10.5	< 10.5 – ≥ 10.0	< 10.0	
Hemoglobin (Male) g/dl	< LLN – ≥ 12.5	< 12.5 – ≥ 11.0	< 11.0	
WBC Increase cell/mm^3	> ULN – < 15,000	≥ 15,000 – < 20,000	≥ 20,000	
WBC Decrease cell/mm^3	< LLN – ≥ 2,500	< 2,500 – ≥ 1,500	< 1,500	
Lymphocytes Decrease cell/mm^3	< LLN – ≥ 750	< 750 – ≥ 500	< 500	
Neutrophils Decrease cell/mm^3	< LLN – ≥ 1,500	< 1,500 – ≥ 1,000	< 1,000	
Platelets Decreased cell/mm^3	< LLN – ≥ 75,000	< 75,000 – ≥ 50,000	< 50,000	

17.2 Appendix II: Case Definitions Acute Myocarditis / Pericarditis

Case Definition for Acute Myocarditis

A possible case of acute myocarditis is defined by the following criteria and the absence of evidence of any other likely cause of symptoms:

Presence of dyspnea, palpitations, or chest pain of probable cardiac origin in a subject with either one of the following:

- ECG abnormalities beyond normal variants, not documented previously, including
- ST-segment or T-wave abnormalities,
- Paroxysmal or sustained atrial or ventricular arrhythmias,
- AV nodal conduction delays or intraventricular conduction defects, or
- Continuous ambulatory electrocardiographic monitoring that detects frequent atrial or ventricular ectopy, **or**
- Evidence of focal or diffuse depressed left-ventricular (LV) function of indeterminate age identified by an imaging trial (e.g., echocardiography or radionuclide ventriculography).

A probable case of acute myocarditis, in addition to the above symptoms and in the absence of evidence of any other likely cause of symptoms, has one of the following:

- Elevated cardiac enzymes, specifically, abnormal levels of cardiac troponin I, troponin T, or creatine kinase myocardial band (a troponin test is preferred);
- Evidence of focal or diffuse depressed LV function identified by an imaging trial (e.g., echocardiography or radionuclide ventriculography) that is documented to be of new onset or of increased degree of severity (in the absence of a previous trial, findings of depressed LV function are considered of new onset if, on follow-up studies, these findings resolve, improve, or worsen); or
- Abnormal result of cardiac radionuclide imaging (e.g., cardiac magnetic resonance imaging with gadolinium or gallium-67 imaging) indicating myocardial inflammation.

A case of acute myocarditis is confirmed if histopathologic evidence of myocardial inflammation is found at endomyocardial biopsy or autopsy.

Case Definition for Acute Pericarditis

A possible case of acute pericarditis is defined by the presence of

- Typical chest pain (i.e., pain made worse by lying down and relieved by sitting up and/or leaning forward) and no evidence of any other likely cause of such chest pain.

A probable case of acute pericarditis is a possible case of pericarditis, or a case in a person with pleuritic or other chest pain not characteristic of any other disease, that, in addition, has one or more of the following:

- Pericardial rub, an auscultatory sign with one to three components per beat,
- ECG with diffuse ST-segment elevations or PR depressions without reciprocal ST depressions that are not previously documented, or
- Echocardiogram indicating the presence of an abnormal collection of pericardial fluid (e.g., anterior and posterior pericardial effusion or a large posterior pericardial effusion alone).

A case of acute pericarditis is confirmed if histopathologic evidence of pericardial inflammation is evident from pericardial tissue obtained at surgery or autopsy.

17.3 Appendix III: Interpretation Support for Assessment of Screening ECGs

For a clearer and mutual understanding of inclusion criterion #14, the following provides clarifying explanations and examples pertaining to eligibility for enrollment.

Examples of subjects **eligible for enrollment**:

- Non-specific ST and T wave changes are not considered clinically significant and subject can be enrolled.
- Sinus bradycardia which does not require clinical intervention is not considered clinically significant and subject can be enrolled.
- Subjects who present with atrial disease which do not require clinical intervention, e.g. a pacemaker or drug treatment are allowed to be enrolled, as these can be considered not clinically significant. Examples are premature atrial contractions or ectopic atrial beats
- Occasional premature ventricular contractions (PVCs) which do not require clinical intervention are not considered clinically significant and subject can be enrolled.
- First degree AV block or PR interval prolongations are also acceptable as long as they do not require clinical intervention, i.e. do not represent an indication for a pacemaker, and therefore the condition can be classified as not clinically significant.
- Right or left axis deviation which does not require clinical intervention is not considered clinically significant and subject can be enrolled.
- QTc prolongations < 500 ms which do not require clinical intervention are not considered clinically significant and subject can be enrolled. QTc prolongations > 500 ms which do not require clinical intervention should be discussed with the Medical Monitor before enrollment.

Examples of subjects **NOT eligible for enrollment**:

- Second or third degree atrioventricular block could represent significant heart disease and subject should not be enrolled.
- Incomplete left bundle branch blocks could represent significant heart disease and subject should not be enrolled.
- Significant ventricular disease represented by complete intraventricular conduction defects (complete left or right bundle branch block) must be considered clinically significant and subjects presenting with any such condition should not be enrolled. Left anterior or posterior intraventricular fascicular blocks or hemiblock could represent ventricular disease and subject should not be enrolled.

ST elevation consistent with ischemia, subject should not be enrolled.

Two premature ventricular contractions (PVCs) in a row, subject should not be enrolled.

17.4 Appendix IV: Grading Scale for Lymphadenopathy

A grading scale for lymphadenopathy would apply as follows:

Grade 0 (normal finding):	No palpable lymph nodes or lymph nodes up to a diameter of 1cm, soft, non-tender
Grade 1 (mild):	Slightly palpable lymph nodes or lymph nodes up to a diameter of 1cm, bilaterally enlarged lymph nodes, signs of tenderness
Grade 2 (moderate):	Markedly palpable lymph nodes or lymph node diameter exceeds 2cm, bilaterally enlarged lymph nodes, pain, skin redness, warmth, limiting instrumental daily life activities
Grade 3 (severe):	Markedly palpable lymph nodes or lymph node diameter exceeds 2cm, generalized enlargement of lymph nodes, sever pain, general symptoms like fever and sweating limiting self care daily activities

17.5 Appendix V: Amendment #1

POX-MVA-013

A Randomized, Double-Blind, Placebo-Controlled Phase III Trial to Evaluate Immunogenicity and Safety of Three Consecutive Production Lots of IMVAMUNE® (MVA-BN®) Smallpox Vaccine in Healthy, Vaccinia-Naïve Subjects

Amendment #1 to Clinical Trial Protocol dated 12-Oct-2012

Date of Amendment #1: 14-Aug-2013

17.5.1 Rationale

After enrolment into the trial started, questions from clinical trial sites have been received asking for clarification on handling trial procedure 'Check In/exclusion criteria' at Visit 3 in conjunction with the procedure for withdrawal from second vaccination. Clarification has been provided to the clinical trial sites via a 'letter to the investigator' including the commitment that a protocol amendment will be initiated to formally capture this clarifications and to remove the unclear wording from the protocol.

During preparation of this amendment the whole protocol has been reviewed and updated for formal consistency.

One discrepancy noted during the review concerns the reporting procedure for serious adverse events (SAEs) and adverse events of special interest (AESIs). In the original trial procedure set up it was planned to report SAEs and AESIs electronically via the eCRF. The electronic reporting procedure could not be implemented, i.e. from trial start and throughout the trial paper forms have been used to report these events. The respective section of the protocol has been updated accordingly.

17.5.2 Changes

General Changes:

- Review and update of the protocol for clarity and formal consistency
- Update of section 1.4 Responsibilities
- Update of Safety Section 8. 3 Reporting to describe process as implemented

Changes have been made as follows:

Changes/ added terms are highlighted in **bold** letters in the text, removed terms are ~~strikethrough~~.

**Clinical Trial Protocol edition #1.0, dated 12-Oct-2012**<br><br>**Previously written:**	**Clinical Trial Protocol edition #2.0, dated 14-Aug-2013**<br><br>**Changed to:**
**Page 13, 1.5 Protocol Synopsis, Vaccination dose**	**Page 13, 1.5 Protocol Synopsis, Vaccination dose**
Two vaccinations four weeks apart (at Day 0 and Day 28) with either 0.5 ml IMVAMUNE® vaccine containing ~~at least~~ 1 x 10^8 $TCID_{50}$ (standard dose) or 0.5 ml placebo (Tris-buffered saline, TBS).	Two vaccinations four weeks apart (at Day 0 and Day 28) with either 0.5 ml IMVAMUNE® vaccine containing **a nominal titer of** 1 x 10^8 $TCID_{50}$ (standard dose) or 0.5 ml placebo (Tris-buffered saline, TBS).<br><br>**Reason for change:** Modified for consistency across protocol.

<table>
<tr><td>Page 19, 1.6Trial Procedure Schedule</td><td>Page 19, Trial Procedure Schedule</td></tr>
<tr><td>
<table>
<tr><th>Visit (V)</th><th>SCR</th><th>V1</th><th>V2</th><th>V3</th></tr>
<tr><td>Check incl. / excl. criteria</td><td>X</td><td>X</td><td></td><td>~~X~~</td></tr>
</table>
</td><td>
<table>
<tr><th>Visit (V)</th><th>SCR</th><th>V1</th><th>V2</th><th>V3</th></tr>
<tr><td>Check incl. / excl. criteria</td><td>X</td><td>X</td><td></td><td></td></tr>
<tr><td>Check withdrawal criteria</td><td></td><td></td><td></td><td>X</td></tr>
</table>

Reason for change: Clarification in line with change on page 33, Visit 3.
</td></tr>
<tr><td>Page 19, 1.6 Trial Procedure Schedule</td><td>Page 19, 1.6 Trial Procedure Schedule</td></tr>
<tr><td>Vaccine administration
& Subject observation (> 30 minutes)</td><td>Vaccine administration
& Subject observation (≥ 30 minutes)

Reason for change: Modified for consistency across protocol.</td></tr>
<tr><td>Page 28, 2.9.1 Potential Risks</td><td>Page 28, 2.9.1 Potential Risks</td></tr>
<tr><td>Trial center staff will watch subjects for 30 minutes after each vaccination and in the event that a severe allergic reaction might occur,</td><td>Trial center staff will watch subjects for at least 30 minutes after each vaccination and in the event that a severe allergic reaction might occur,

Reason for change: Modified for consistency across protocol.</td></tr>
<tr><td>Page 30, 4.2.2. Active Trial Phase</td><td>Page 31, 4.2.2. Active Trial Phase</td></tr>
<tr><td>At Visit 1 / Day 0, subjects will receive the first of two s.c. vaccinations with one standard dose of IMVAMUNE® (0.5 ml vaccine containing 1 x 10^8 $TCID_{50}$) or placebo containing TBS in the non-dominant upper arm (deltoid region).</td><td>At Visit 1 / Day 0, subjects will receive the first of two s.c. vaccinations with one standard dose of IMVAMUNE® (0.5 ml vaccine containing a nominal titer of 1 x 10^8 $TCID_{50}$) or placebo containing TBS in the non-dominant upper arm (deltoid region).

Reason for change: Modified for consistency across protocol.</td></tr>
<tr><td>Page 31, 4.2.2. Active Trial Phase, Visit 1
Page 32, 4.2.2. Active Trial Phase, Visit 3</td><td>Page 31, 4.2.2. Active Trial Phase, Visit 1
Page 32, 4.2.2. Active Trial Phase, Visit 3</td></tr>
<tr><td>The vaccine can be administered to persons with a minor illness such as diarrhea, mild upper respiratory infection, or any other mild condition with or without low-grade febrile illness, i.e. oral temperature ≤ 100.4°F (≤</td><td>The vaccine can be administered to persons with a minor illness such as diarrhea, mild upper respiratory infection, or any other mild condition with or without low-grade febrile illness, i.e. oral temperature < 100.4°F (<</td></tr>
</table>

38.0°C).	**38.0°C)**.<br><br>**Reason for change:** Modified for consistency across protocol.
**Page 32, 4.2.2. Active Trial Phase, Visit 3**	**Page 32, 4.2.2. Active Trial Phase, Visit 3**
Tasks to be performed prior to vaccination:<br><br>~~• (Re) Check inclusion / exclusion criteria~~	Tasks to be performed prior to vaccination:<br><br>• **Check withdrawal criteria**
**Page 40, 8.1.1 Medical History**	**Page 40, 8.1.1 Medical History**
Symptoms present before ~~or at the screening visit~~ will be documented in the medical history.	Symptoms present before **ICF signature** will be documented in the medical history.<br><br>**Reason for change:** Modified for clarity.**-**
**Page 40, 8.1.2 Baseline Sign and Symptoms**	**Page 40, 8.1.2 Baseline Sign and Symptoms**
Any new signs, symptoms or changes in health that occur after ~~SCR (status recorded as "medical history")~~ and before the first vaccination will be recorded in the baseline signs and symptoms sections of the eCRF and in the subject`s medical record.	Any new signs, symptoms or changes in health that occur after **ICF signature** and before the first vaccination will be recorded in the baseline signs and symptoms sections of the eCRF and in the subject's medical record.<br><br>**Reason for change:** Modified for clarity.**-**
**Page 40, 8.1.3 AE**	**Page 40, 8.1.3 AE**
AEs are recorded based on unsolicited and solicited questioning.	**New signs, symptoms or changes in health starting after the first vaccination are documented in the AE section.** AEs are recorded based on unsolicited and solicited questioning.<br><br>**Reason for change:** Modified for clarity.**-**
**Page 49, 8.2.8 Safety Laboratory Measures**	**Page 49, 8.2.8 Safety Laboratory Measures**
Serum chemistry:<br>Total bilirubin, AP, AST, ALT, serum creatinine (for calculation of CrCl), sodium, potassium, calcium, troponin I (troponin I mandatory at the SCR and Visit 2 and in addition at Visit 4 if clinically indicated).	Serum chemistry:<br>Total bilirubin, AP, AST, ALT, serum creatinine (for calculation of CrCl **at SCR**), sodium, potassium, calcium, troponin I (troponin I mandatory at the SCR and Visit 2 and in addition at Visit 4 if clinically indicated).

	**Reason for change:** Modified to match Laboratory procedures performed.
**Page 54 and 55, 9.3 Sample Size Calculation**	**Page 55 and 56, 9.3 Sample Size Calculation**
(Karjalainen and Heikkilä, 1998)	(Karjalainen and Heikkilä, 1999)<br><br>**Reason for change:** Correction of wrong year of reference.

All changes are considered not to have any negative influence on the trial procedures in general, on the safety of the trial participants or on the validity of the trial result.
